# ALS/FTD Mutation-Induced Phase Transition of FUS Liquid Droplets and Reversible Hydrogels into Irreversible Hydrogels Impairs RNP Granule Function

**DOI:** 10.1016/j.neuron.2015.10.030

**Published:** 2015-11-18

**Authors:** Tetsuro Murakami, Seema Qamar, Julie Qiaojin Lin, Gabriele S. Kaminski Schierle, Eric Rees, Akinori Miyashita, Ana R. Costa, Roger B. Dodd, Fiona T.S. Chan, Claire H. Michel, Deborah Kronenberg-Versteeg, Yi Li, Seung-Pil Yang, Yosuke Wakutani, William Meadows, Rodylyn Rose Ferry, Liang Dong, Gian Gaetano Tartaglia, Giorgio Favrin, Wen-Lang Lin, Dennis W. Dickson, Mei Zhen, David Ron, Gerold Schmitt-Ulms, Paul E. Fraser, Neil A. Shneider, Christine Holt, Michele Vendruscolo, Clemens F. Kaminski, Peter St George-Hyslop

**Affiliations:** 1 Tanz Centre for Research in Neurodegenerative Diseases, and Departments of Medicine, Medical Biophysics and Laboratory Medicine and Pathobiology, University of Toronto, Toronto, Ontario M5S 3H2, Canada; 2 Cambridge Institute for Medical Research, Cambridge National Institute for Health Research - Biomedical Research Unit in Dementia, University of Cambridge, Cambridge CB2 0XY, UK; 3 Department of Physiology, Development, and Neuroscience, University of Cambridge, Cambridge CB2 3DY, UK; 4 Department of Chemical Engineering and Biotechnology, University of Cambridge, Cambridge CB2 3RA, UK; 5 Department of Chemistry, University of Cambridge, Cambridge, CB2 1EW, UK; 6 Centre for Genomic Regulation and University Pompeu Fabra, Dr. Aiguader St. 88, and Universitat Pompeu Fabra, 08003, Barcelona, Spain; Institució Catalana de Recerca i Estudis Avançats (ICREA), 23 Passeig Lluís Companys, 08010 Barcelona, Spain; 7 Cambridge Systems Biology Center & Department of Biochemistry, University of Cambridge, 80 Tennis Court Road, Cambridge CB2 1GA, UK; 8 Department of Research, Neuroscience, Mayo Clinic College of Medicine, 4500 San Pablo Road, Jacksonville, FL 32224, USA; 9 Samuel Lunenfeld Research Institute, Mount Sinai Hospital, and Department of Molecular Genetics, University of Toronto, Toronto, Ontario M5G 1X5, Canada; 10 Department of Clinical Biochemistry, Cambridge Institute for Medical Research, University of Cambridge, Cambridge CB2 0XY, UK; 11 Department of Neurology, Center for Motor Neuron Biology and Disease, Columbia University Medical Center, New York, NY 10032, USA

## Abstract

The mechanisms by which mutations in FUS and other RNA binding proteins cause ALS and FTD remain controversial. We propose a model in which low-complexity (LC) domains of FUS drive its physiologically reversible assembly into membrane-free, liquid droplet and hydrogel-like structures. ALS/FTD mutations in LC or non-LC domains induce further phase transition into poorly soluble fibrillar hydrogels distinct from conventional amyloids. These assemblies are necessary and sufficient for neurotoxicity in a *C. elegans* model of FUS-dependent neurodegeneration. They trap other ribonucleoprotein (RNP) granule components and disrupt RNP granule function. One consequence is impairment of new protein synthesis by cytoplasmic RNP granules in axon terminals, where RNP granules regulate local RNA metabolism and translation. Nuclear FUS granules may be similarly affected. Inhibiting formation of these fibrillar hydrogel assemblies mitigates neurotoxicity and suggests a potential therapeutic strategy that may also be applicable to ALS/FTD associated with mutations in other RNA binding proteins.

## Highlights

• FUS phase transitions between monomer, liquid droplet, and hydrogel states
• FUS mutants induce further phase transition into irreversible fibrillar hydrogels
• Irreversible hydrogels sequester RNP cargo and impair RNP granule function
• Formation of non-amyloid fibrillar hydrogels provides a compelling causative mechanism for neurodegeneration

## Teaser

Murakami et al. show that FUS transitions between monomer, liquid droplet, and hydrogel states during uptake and release of RNP granule cargo. FUS mutations accelerate transition into fibrillar hydrogels that trap RNP cargo, impair RNP granule function, and cause neurodegeneration.

## Introduction

Mutations in a growing list of RNA binding proteins (RBPs) including TDP-43, FUS, ataxin2, hnRNPA1, and B2, are associated with sporadic and inherited forms of amyotrophic lateral sclerosis and frontotemporal dementia (hereafter “ALS/FTD”) (van der Zee and Van Broeckhoven, 2014). A neuropathological hallmark of these disorders is the deposition of poorly soluble assemblies of the mutant RBP in the nucleus and cytoplasm of neurons in the brain and spinal cord of ALS/FTD patients.

The pathogenic significance of these intraneuronal assemblies has been the subject of much speculation. Hypotheses have included that they might (i) be irrelevant secondary phenomena, (ii) form conventional neurotoxic amyloids, or (iii) bind and trap crucial RNA sequences (Couthouis et al., 2011, Gendron et al., 2010, Neumann et al., 2007, Rademakers et al., 2012, Ticozzi et al., 2010). Although there are data to support the notion that these aggregates may be amyloid like (Fang et al., 2014, Robinson et al., 2013), other evidence suggests that they are distinct from classical amyloids (Cairns et al., 2007, Dormann et al., 2009, Murakami et al., 2012). Specifically, we and others have shown that in contrast to classical amyloids, pathological RBP deposits are (1) soluble in urea, (2) have low β sheet content, (3) have a mixed granular/fibrillar appearance under EM, (4) do not bind amyloidophyllic dyes (e.g., Thioflavin T), and (5) when fluorescently labeled, do not display the marked reductions in in vivo fluorescent lifetimes that are typical of conventional amyloids (Kaminski Schierle et al., 2011) (Figures S1–S3).

Recently, attention has refocused on the possibility that mutant ALS/FTD-associated RBPs might be incorporated into and alter the function of ribonucleoprotein (RNP) granules (Li et al., 2013, Mitchell and Parker, 2014, Ramaswami et al., 2013, Wolozin, 2012). However, these hypotheses have not been directly proven.

To investigate this possibility, we utilized FUS as a tractable example of the RBP-proteinopathies. We describe here the results of an extensive array of biophysical, biochemical, cellular, and animal model experiments conducted over the past 4 years. In partial agreement with earlier reports (Han et al., 2012, Kato et al., 2012), we show that in a process driven by the LC domain at the N terminus of FUS, FUS undergoes phase transitions, reversibly shifting between dispersed, liquid droplets, and hydrogel-like phases. Crucially, we demonstrate that pathogenic FUS mutants severely limit this ability to reiteratively shift between these phases. In fact, FUS mutants increase the propensity of FUS to condense into poorly soluble, stable, fibrillar hydrogel-like assemblies. We show that these stable “irreversible” FUS assemblies: selectively entrap other ribonucleoproteins, impair local RNP granule function, and attenuate new protein synthesis in the axon terminals of cultured neurons. We do not exclude the possibility that formation of fibrillar hydrogels may have similar disruptive effects on nuclear RNP granules. Importantly, we show that modulation of these protein phase transitions may be a tractable therapeutic strategy.

## Results

**Figure 1:**
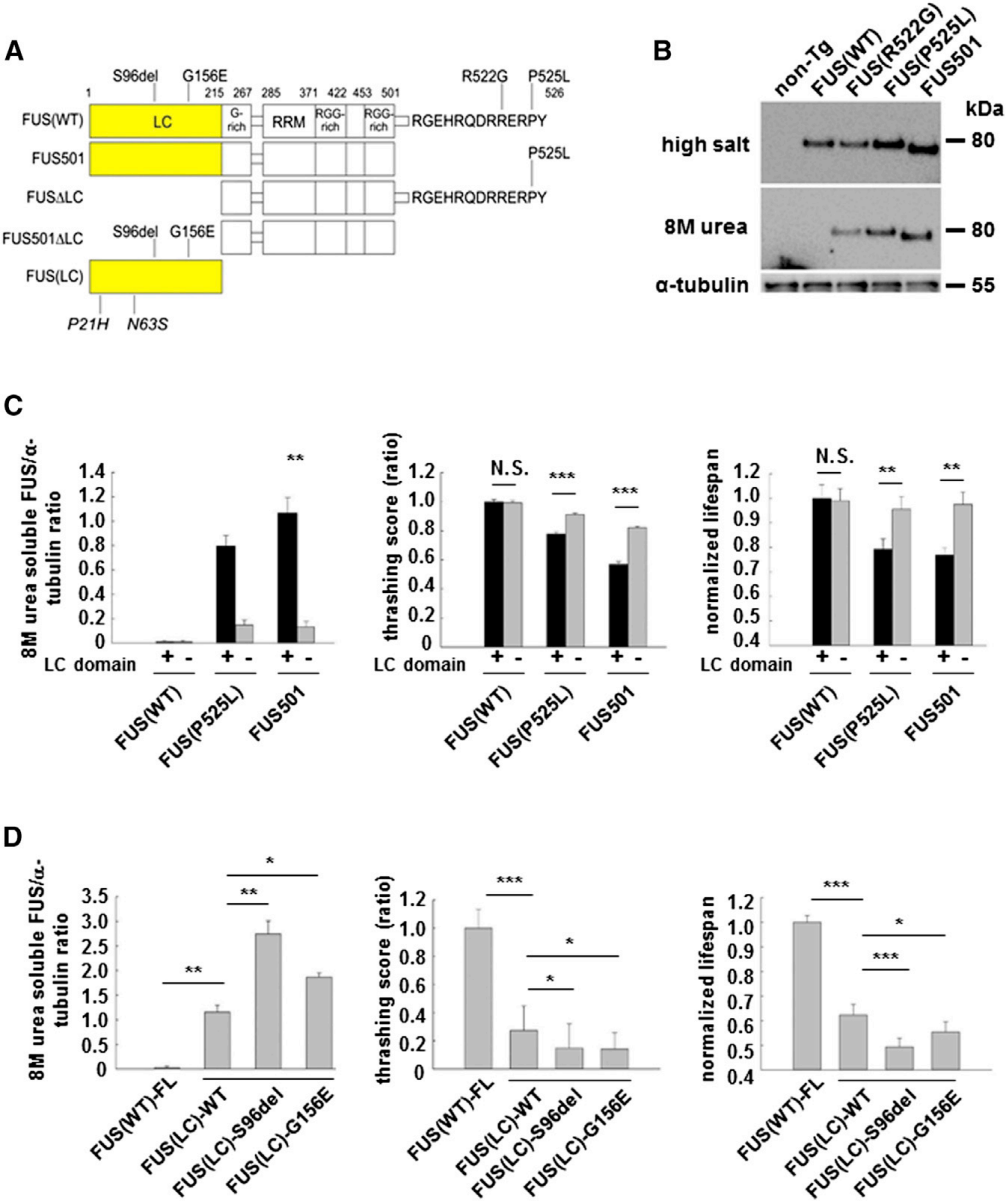
FUS Constructs, Cellular Localization, and Neurotoxicity of Mutant FUS (A) FUS constructs and mutations. Full-length FUS constructs include wild-type FUS(WT) and ALS/FTD mutations (R522G, R524S, P525L, and R495X), or FUS (FUS501), which mimics several ALS/FTD truncation mutants. LC motif at residues 2–214 was deleted in FUS(WT)ΔLC, FUS(P525L)ΔLC, and FUS501ΔLC. FUS(LC)-only constructs include wild-type FUS(LC), pathogenic S96del or G156E mutants, or clinically benign P21H and N63S sequences. LC, low-complexity motif; G-Rich, glycine-rich region; RRM, RNA recognition motif; RGG-rich, arginine-glycine-glycine-rich region. (B) FUS(WT) is located in neuronal nuclei; mutant FUS accumulates as 8 M urea-soluble assemblies in neuronal cytoplasm. Western blot of high-salt buffer extracted whole-worm lysates (top), 8 M urea extracted lysates (middle), and α-tubulin (bottom). (C) FUS LC domain is necessary and sufficient for FUS assembly and neurotoxicity. Deletion of LC domain from FUS(P525L) and FUS 501 reduces formation of 8 M urea-soluble FUS (left panel), improves motor function (middle panel), and lifespan in mutant FUS lines (right panel), but has no impact on FUS(WT) animals. N.S., not significant. ^∗∗^p < 0.01; ^∗∗∗^p < 0.001. (D) FUS LC domain is necessary and sufficient for FUS assembly and neurotoxicity. Overexpression of wild-type FUS(LC) alone is associated with increased 8 M urea-soluble assemblies (left panel), impaired motor function (middle panel), and lifespan (right panel). The ALS/FTD FUS(LC)-mutations (S96del and G156E) further augment neurotoxicity. ^∗^p < 0.05, ^∗∗^p < 0.01, and ^∗∗∗^p < 0.001. Error bars are SEM.

To rigorously test the idea that mutations in FUS alter its assembly into RNP granules, we first explored the biology of FUS in a *C. elegans* model of FUS proteinopathy (Murakami et al., 2012). In this model, equivalent levels of wild-type or mutant human FUS protein are expressed under the neuron-specific *Prgef-1* promoter with a GFP tag, which does not alter the activity or location of FUS (Murakami et al., 2012). We focused on animals (n ≥ 3 lines per mutant) expressing wild-type FUS (FUS(WT)) or one of five ALS/FTD mutants: FUS (R495X), FUS(R522G), FUS(R524S), FUS(P525L), and FUS501(which models several ALS/FTD-associated C-terminal truncating mutations) (Figure 1A). These mutations span a wide range of predicted aggregation propensities as calculated using the Zyggregator method (Tartaglia and Vendruscolo, 2008): FUS(R495X): Zagg = 0.44; FUS(R522G): Zagg = 0.09; FUS(R524S): Zagg = 0.05; FUS(P525L): Zagg = 0.12; FUS501: Zagg = 0.52 (Figure S4). The aggregation propensities of these mutants correlate with their phenotypic severities in *C. elegans* and in humans, ranging from mild (R522G) to severe (FUS (P525L), FUS (R495X), and FUS501) (Murakami et al., 2012) (Figure S5).

This *C. elegans* model displays several features observed in human FUS-related disorders (Murakami et al., 2012). Thus, in animals expressing wild-type FUS, FUS is expressed physiologically in the nucleus, and causes no detectable neurotoxicity (Figures S6 and S7). In contrast, animals expressing mutant FUS display age-dependent neurotoxicity manifested by impaired motor function and reduced lifespan (Figures S7 and S8). These phenotypes are accompanied by nuclear and cytoplasmic deposits of FUS that are insoluble in high-salt and RIPA buffers but are soluble in 8 M urea (Figure 1B). The ex vivo abundance of these 8 M urea-soluble species correlates with the in vivo abundance of visible cytoplasmic FUS assemblies in neurons (r^2^ = 0.96) (Figure S8). As is the case in human brain expressing mutant FUS, these pathological FUS assemblies are biochemically and biophysically distinct from conventional neurotoxic amyloids (Figures S1–S3).

### Non-Amyloid 8 M Urea-Soluble FUS Assemblies Cause Neurotoxicity

Two lines of evidence indicate that FUS assemblies drive neurotoxicity. First, in animals expressing equivalent quantities of total FUS protein, the abundance of 8 M urea-soluble FUS assemblies correlates directly with neurotoxicity as measured by motor activity (r^2^ = 0.69) and lifespan (r^2^ = 0.88) (Figure S7). Second, within each line, neurotoxicity can be increased or decreased by manipulating the abundance of the 8 M urea-soluble assemblies. In lines expressing ALS/FTD-associated mutant FUS, increasing the abundance of these 8 M urea-soluble assemblies (e.g., by heat-stress-induced depletion of molecular chaperones) exacerbated motor deficits and accelerated mortality (Figure S9; p < 0.01; n = 16 animals/ strain). Conversely, reducing the abundance of 8 M urea-soluble assemblies, either by treatment with protein aggregation inhibitors such as scyllo-inositol, or by overexpression of hsp-70, rescued both motor function and mortality of mutant FUS strains but not in non-transgenic N2 or FUS(WT) animals (Figure S10; *scyllo-inositol: p <* 0.01; n = 20 animals/strain; *hsp-70*: p < 0.001, n = 20 animals/strain).

### Neurotoxicity Is Driven by the FUS Low-Complexity Domain

The N-terminal low-complexity (LC) domain of FUS (residues 2–214) has been reported to induce the assembly of wild-type FUS into hydrogel-like assemblies (Han et al., 2012, Kato et al., 2012). Consistent with this observation, calculation of the biophysical aggregation propensity of FUS revealed that the LC domain is highly prone to aggregate. However this tendency is offset by the lower aggregation propensity of the C-terminal domains (Figure S4). Taking these observations together, we hypothesized that (1) ALS/FTD mutations in the FUS LC domain (e.g., S96del and G156E) might directly increase the propensity of the LC domain to induce FUS assembly into higher order structures; while (2) ALS/FTD mutants in the C terminus would indirectly have the same effect by reducing the anti-aggregation effect of the C terminus (Figure S4).

In agreement with these predictions, animals expressing mutant FUS constructs lacking the LC domain had significantly less 8 M urea-soluble assemblies and less neurotoxicity (Figure 1C; n = 20 animals/strain; p < 0.01 for 8 M urea FUS and lifespan; p < 0.001 for thrashing score; Figure S11). Conversely, expression of FUS(LC) alone induced robust 8 M urea-soluble assemblies accompanied by increased motor deficits and mortality (Figure 1D, p < 0.01, Figure S12; p < 0.001; n = 20 animals/line). As predicted, the severity of these FUS(LC)-induced phenotypes was further enhanced in animals expressing FUS(LC) domain containing the ALS/FTD associated mutants: S96del or G156E (Figure 1D; p < 0.05; n = 20 animals /line, 3 replications; Figure S12).

### Manipulation of Other Components of FUS-Containing RNP Granules Rescues Toxicity

**Figure 2:**
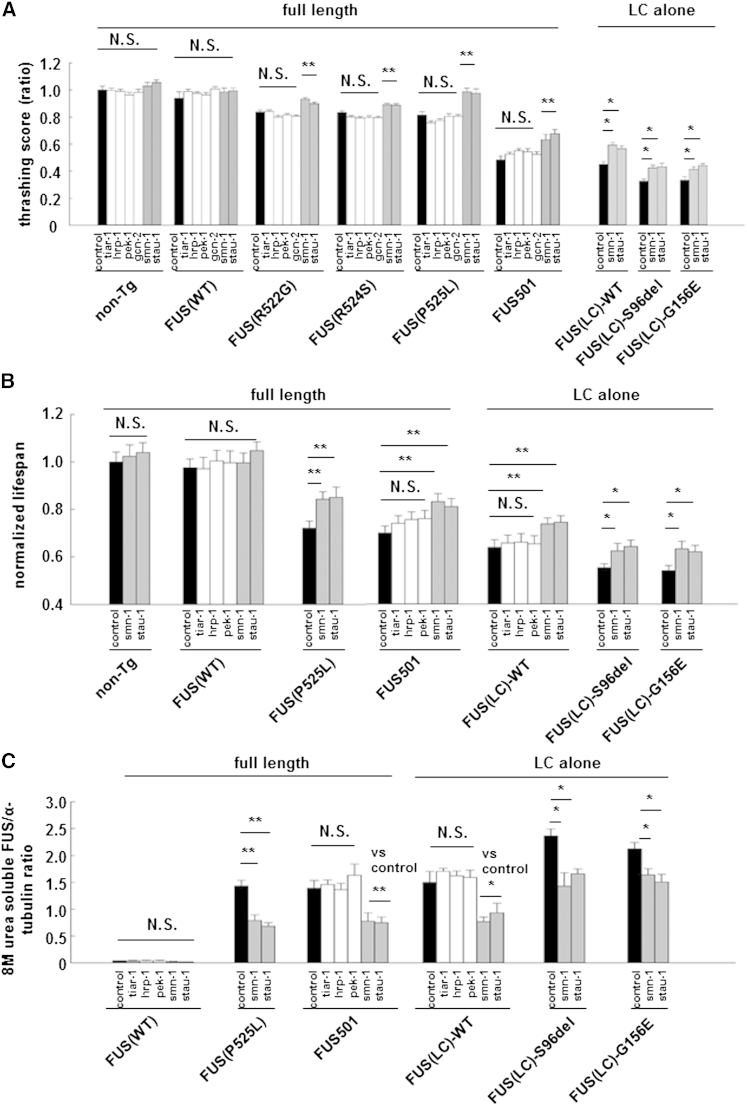
Selected RNA Binding Proteins Co-Localized with FUS in Mutant FUS Assemblies and Their RNAi Knockdown Partially Rescued Neurotoxicity (A) A directed RNAi screen of selected LC-domain containing RBPs revealed that RNAi knockdown of SMN (smn-1) and STAU-1 (stau-1) (gray bars) partially rescued motor function, whereas control RNAi knockdown (black bars) and RNAi knockdown of TIA-1 (tiar-1), hnRNP (hrp-1), EIF2AK (pek-1), and EIF2AK3 (gcn-2) (white bars) had no effect (N.S, not significant; ^∗^p < 0.05 and ^∗∗^p < 0.01). (B) RNAi knockdown of SMN and STAU-1 (gray bars) rescued lifespan, whereas RNAi of the other RNPs (white bars) and control RNAi (black bars) had no effect. N.S., not significant. ^∗^p < 0.05 and ^∗∗^p < 0.01. (C) RNAi knockdown of SMN and STAU-1 (gray bars) reduced 8 M urea-soluble FUS assemblies whereas control RNAi (black bars) and RNAi of the other RNPs (white bars) had no effect. N.S: not significant. ^∗^p < 0.05; ^∗∗^p < 0.01. Error bars are SEM.

Pathological FUS assemblies contain other components of RNP granules including other RNA-binding proteins (Li et al., 2013, Mitchell and Parker, 2014, Ramaswami et al., 2013, Wolozin, 2012). We wondered whether some of these other components may stabilize FUS assemblies, as is the case with many other heteromeric complexes. If so, then knockdown of a subset of these RNP granule components might destabilize the pathological complexes and ameliorate neurotoxicity. We selected seven RNPs that (1) are components of RNP granules, (2) have human and *C. elegans* homologs, and (3) have validated neuron-specialized RNAi clones available to knock down the *C. elegans* homolog. This list comprised CBP (cbp-1), EIF2AK (pek-1), EIF2AK3 (gcn-2), STAU-1 (stau-1), SMN (smn-1), hnRNP1 (hrp-1), and TIA-1 (tiar-1) (upper case = human; lower case = *C. elegans* ortholog). RNAi of cbp-1 was uninterpretable because it caused non-specific effects in non-transgenic animals. RNAi of tia-1, hrp-1, gen-2, and pek-1 did not alter thrashing scores, lifespan, or abundance of 8 M soluble FUS levels in animals expressing FUS(WT), mutant FUS, or FUS(LC) (white bars in Figures 2A–2C; p = N.S). In contrast, RNAi knockdown of stau-1 or smn-1 partially rescued neurotoxicity, reduced 8 M urea-soluble FUS assemblies, and reduced visible cytoplasmic FUS deposits in all mutant FUS strains tested (gray bars in Figures 2A–2C and S13; p < 0.05, n = 20 animals/strain, three replications).

**Figure 3:**
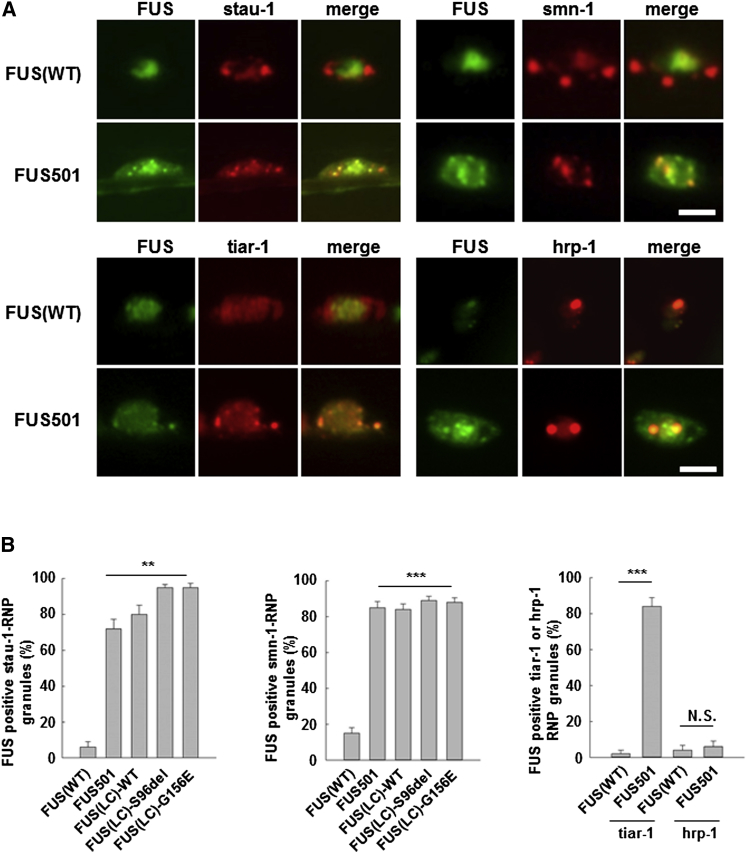
STAU-1, SMN, and TIAR-1 but Not hnRNP1 Colocalize with Mutant FUS in Cytoplasmic RNP Granules STAU-1, SMN, and TIAR-1 (red) colocalized with pathological intraneuronal cytoplasmic and axonal assemblies of mutant full-length FUS501, wild-type FUC-LC domain and mutant FUS(LC) domain (green) but did not colocalize with nuclear FUS(WT) (green). ^∗∗∗^p < 0.001. Scale bars, 2.0 μm. Additional data are shown in Figures S3B–S3D. *STAU-1* colocalization: FUS(WT) = 6.0% ± 3.1%; FUS501 = 72.0 ± 5.3%; FUS(LC) = 80.0 ± 5.2%; FUS(LC)-S96del = 95.0 ± 1.7%; FUS(LC)-G156E = 95.0 ± 2.2; n = 20 neurons; p < 0.001; *SMN* colocalization: (FUS(WT) = 15.0 ± 3.1%; FUS 501 = 85.0 ± 3.4%; FUS(LC) = 84.0 ± 3.1%; FUS(LC)-S96del = 89.0 ± 2.3%; FUS(LC)-G156E = 88.0 ± 2.5%; n = 20 neurons; p < 0.001). Scale bars, 2.0 μm. Error bars are SEM.

To determine whether the rescue effect was mediated at the level of FUS granules, we co-expressed RFP-tagged STAU-1 or SMN in animals expressing various FUS cDNAs. STAU-1 and SMN strongly colocalized (≥72% ± 5.3% overlap) with cytoplasmic assemblies comprised of mutant full-length FUS or aggregation-prone FUS(LC) domain. STAU-1 and SMN did not colocalize with FUS(WT) (6.0% ± 3.1%; Figures 3D and S14; n = 20 neurons; p < 0.001).

The colocalization of STAU-1 and SMN with mutant 8 M urea-soluble FUS granules was semi-specific. Thus hnRNP1, which has no effect on FUS toxicity (Figures 2A–2C), poorly colocalized with mutant FUS assemblies (Figure 3; hnRNP1 colocalization: FUS(WT) and FUS501 ≤ 6.0% ± 3.1%; p = N.S.). We have reported similar lack of colocalization for PAB-1 (Murakami et al., 2012). Intriguingly, TIAR-1, which is a known component of stress granules, did colocalize with pathological cytoplasmic FUS assemblies, and as described below, its presence in mutant FUS granules may have functional significance (TIAR-1 colocalization: FUS(WT) = 2.0% ± 2.0%; FUS501 = 84.0% ± 5.0%; p < 0.001) (Figure 3). Taken together, these results suggest that pathological FUS assemblies selectively trap other RNPs. This conclusion led us to ask two further questions. First, could physiological FUS assemblies form reversible structures that transiently sequester RNP cargo upon gel-formation, but then release it upon melting? Second, do irreversible FUS assemblies permanently trap their RNP cargo?

### FUS LC Domain Induces Reversible Liquid Droplet and Hydrogel Formation

**Figure 4:**
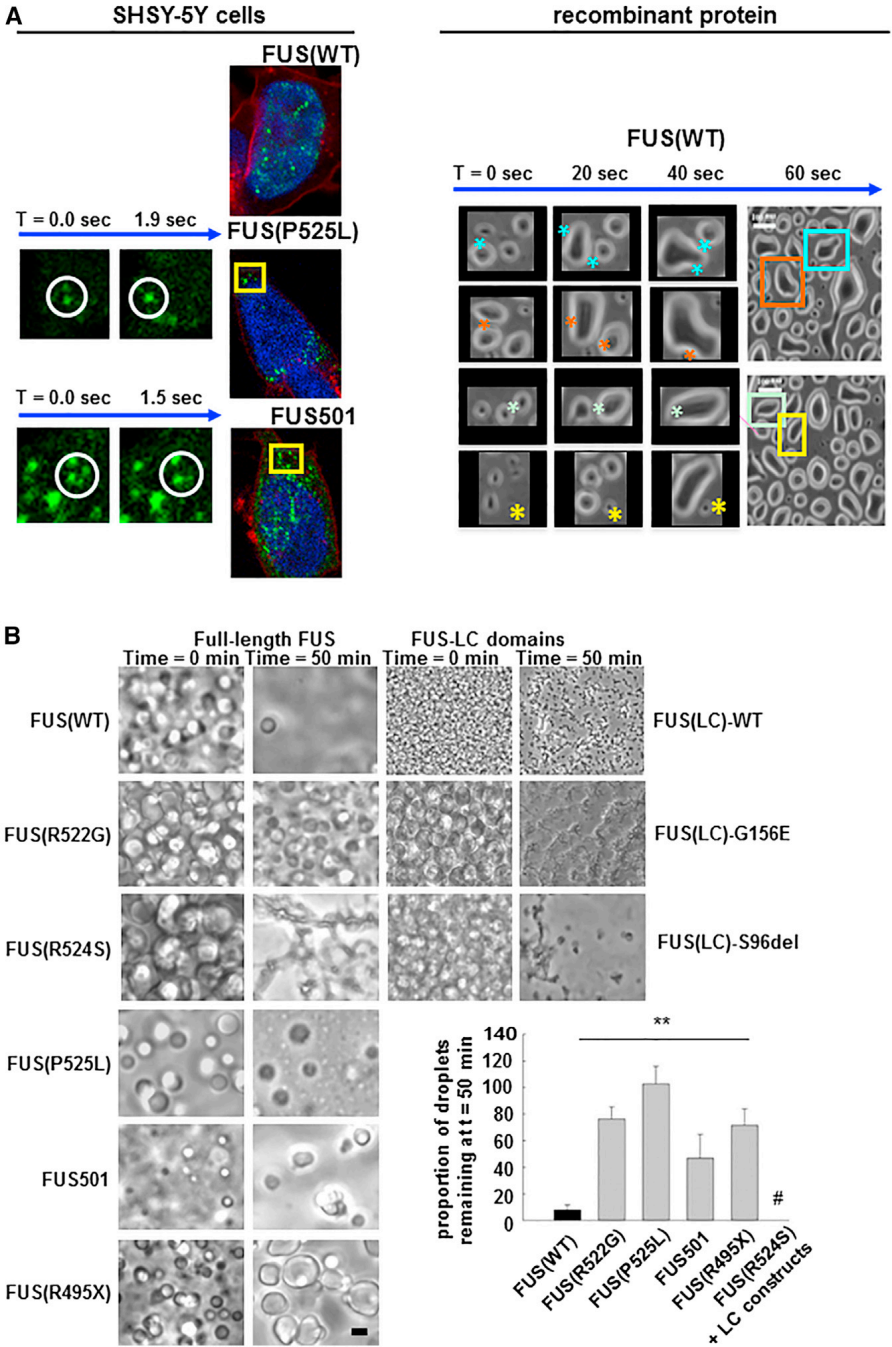
Recombinant FUS Generates Liquid Droplet-Like Assemblies, the Reversibility of which Is Reduced by ALS/FTD Mutants (A) (Left panel) SHSY5Y cells transiently transfected with YFP-tagged wild-type FUS show static intranuclear spherical structures. (Top) In the same cell type, mutant FUS localizes in the cytoplasm as mobile spherical structures (“droplets”) that move, fuse, and disappear (middle and bottom—time series of two fusion events). (Right panel) Cooling of 1–5 μM solutions of either full-length FUS or FUS(LC) domain, induces spontaneous phase transition into liquid droplets, which spontaneously fuse (^∗^), split over seconds, and redisperse into solute over ∼50 min. (B) Full-length FUS(WT) liquid droplets are more labile than full-length mutant FUS or FUS(LC) droplets. Both mutant and wild-type FUS proteins form multiple bright spherical liquid droplets after cooling (left column). FUS(WT) droplets almost completely disperse by 50 min at 23°C (right column). FUS proteins containing ALS/FTD mutants formed more stable droplets, many of which were still visible at 50 min at 23°C. The residual mutant assemblies had darkly refractile properties, angular edges, and/or spiculated appearances. We interpret these structures to represent further phase transitions to gel-like or solid assemblies. Spiculated assemblies were especially prevalent with the FUS(LC) constructs, where virtually all of the residual FUS(LC) material had this appearance (# in the graph). Error bars are SEM.

To address these questions, we began by exploring the impact of FUS mutations on RNP granule formation in SHSY5Y mammalian neuronal cells transiently transfected with YFP-tagged wild-type FUS, P525L, or FUS501 constructs. As was the case in vivo in our *C. elegans* model, in cells expressing YFP-tagged wild-type FUS, FUS was predominantly localized as spherical intranuclear droplets (Figure 4A, left panel). In cells expressing YFP-tagged mutant FUS, FUS was present as similar structures in the cytoplasm and nucleus. Live cell imaging revealed that the nuclear droplets were static, but the cytoplasmic droplets were motile and on occasion fused with each other in a process previously likened to liquid droplets (Brangwynne et al., 2009) (Figure 4A, left panel).

To investigate the biophysics of FUS assembly into these liquid droplet assemblies, and to test the hypothesis that mutations in FUS might alter the dynamics of this process, we expressed and purified recombinant wild-type full-length FUS and full-length FUS containing one of five different mutations (FUS(R522G), FUS(R524S), FUS(P525L), FUS501, and FUS(G495X)). At 23°C in near-physiological protein and salt concentrations (1–5 μM protein; 150 mM NaCl), all of the FUS proteins were fully dispersed in solution. When cooled to 4°C to reduce thermal motion and thus stabilize weak interactions, they all assembled into visible liquid droplets that frequently fused with each other over timescales of 20–40 s (Figure 4A, right panel). When droplets composed of FUS(WT) were maintained at 23°C, they almost completely dissolved within 50 min (Figure 4B). In contrast, when liquid droplets composed of mutant FUS were treated in the same way, more than 50% of mutant FUS assemblies persisted at 50 min (Figure 4B, p < 0.01 n = 3 replications). Crucially, several of the full-length FUS mutants (especially FUS (R524S)) and all of the LC-domain constructs tended to produce dark, angled, or spiculated structures, which we interpreted to be solid (Figure 4B).These analyses suggest that pathogenic FUS mutations favor phase transitions of FUS from a monomeric dispersed state into a liquid droplet and then into a more stable solid form. These observations were robust but were difficult to quantify in detail because liquid droplet formation is exquisitely sensitive to changes in ionic strength and temperature of the buffer.

### ALS/FTD-Associated Mutations in the FUS LC Domain Alter Hydrogel Properties

**Figure 5:**
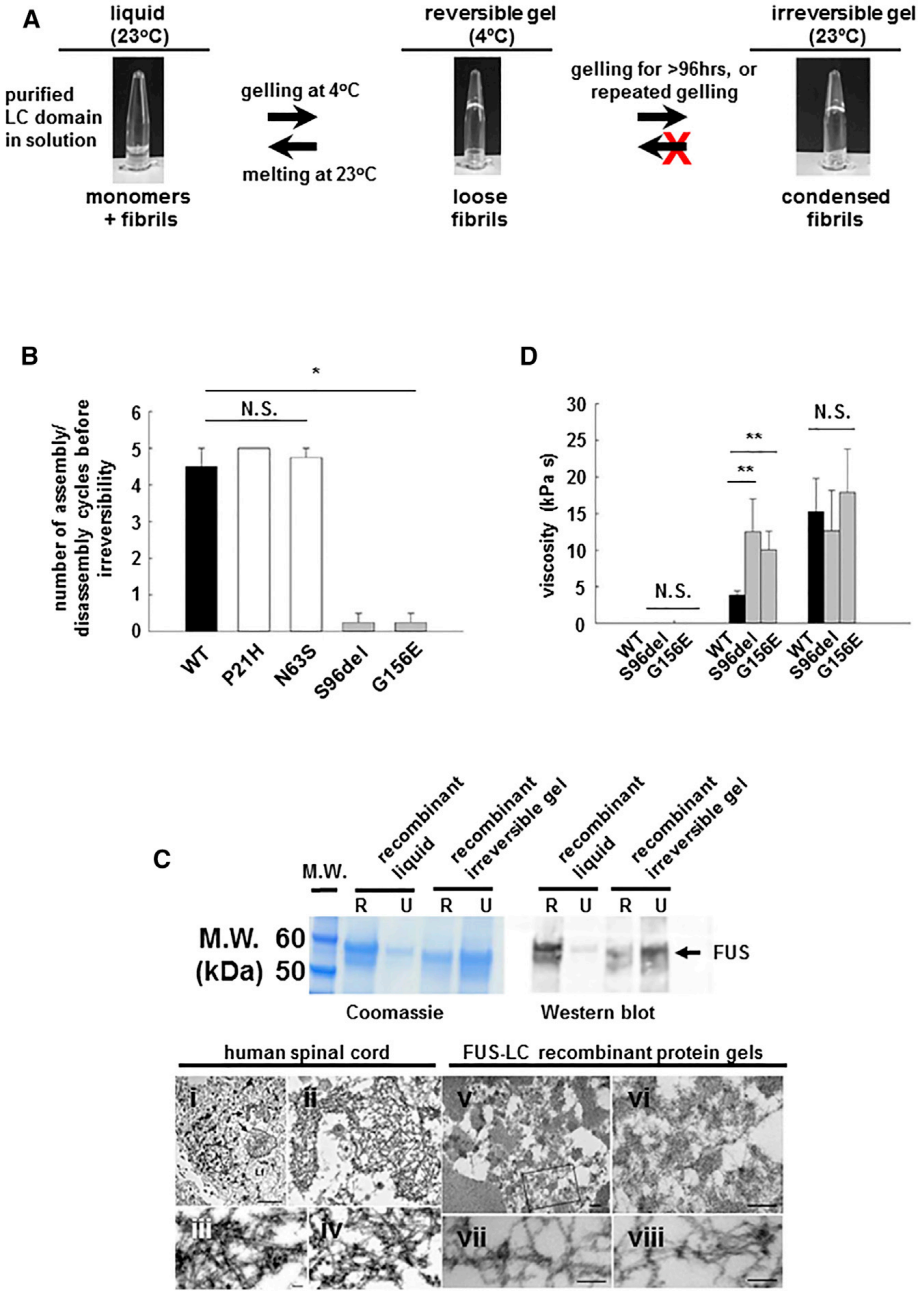
Recombinant FUS Generates Gel-Like Assemblies, the Reversibility of which Is Reduced by ALS/FTD Mutants (A) Purified recombinant FUS(LC) forms clear solution at 23°C (Top left panel) but forms reversible gel-like structures at 4°C (Top center panel). Rewarming to 23°C reverts FUS(LC) to a liquid state (top left panel). Repeated cycling of wild-type FUS(LC) eventually results in the formation of irreversible gels (top right panel). (B) ∼4 to 5 assembly/disassembly cycles can be achieved by wild-type FUS(LC) (black bar) and by FUS(LC) containing benign polymorphic variants (P21H and N63S, white bars). ALS/FTD mutations (gray bars) dramatically reduce the number of cycles to ≤1 before irreversibility (^∗∗^p < 0.01). (C) Top panel: Coomassie gel (left) and western blot (right) of RIPA- and 8 M urea solubilisation of FUS(LC) reveal that liquid state FUS(LC) is fully soluble in RIPA buffer. Irreversible FUS(LC) is partly soluble in RIPA, but fully soluble in 8 M urea. Bottom panel: Transmission electron microscopy of human FUS inclusions ([Ci]–[Civ]) and irreversible recombinant protein gels ([Cv]–[Cviii]). Both are composed of loose networks of ∼14 nm fibrils. Scale bars are 500 nm ([Cv] and [Cvi]) and 100 nm ([Cvii] and [Cviii]). (D) Analysis of liquid, reversible gel, and irreversible gel using fluorescent particle tracking revealed that liquid (left cluster) and reversible gels (black bars, middle cluster) have viscosities similar to those of P-granules. Mutant reversible gels (gray bars, middle cluster) were more viscous than wild-type reversible gels. Irreversible gels, regardless of whether made from wild-type or ALS/FTD mutant FUS exhibit very high viscosities. N.S., not significant; ^∗∗^p < 0.01. Error bars are SEM.

To quantitatively assess the biophysical features that govern phase transition of FUS from soluble monomer to reversible liquid droplet and then to more stable assemblies, and to assess the consequences of these phase transitions, we adapted a previously published method (Han et al., 2012, Kato et al., 2012) that provides a simple and tractable platform to produce a working model of reversible (physiological) and irreversible (pathological) assembly of FUS into higher-order structures. Briefly, when 0.25–1 mM solutions of wild-type FUS(LC) in 200–500 mM NaCl are cooled to 4°C, they condensed into gels. Upon rewarming to 23°C, the gels re-dissolve into a clear solution (Figure 5A). This liquid→gel→liquid cycle can be assayed by placing the protein solution into a siliconized microtube and then varying the temperature between 4°C and 23°C. Liquid assemblies readily fall under gravity when the tube is inverted. Gelled assemblies remain at the bottom of the tube (Figure 5A).

Using this assay, wild-type FUS(LC) (Figure 5B, black bar, left panel) and FUS(LC) domains containing either of the non-pathogenic variants (P2IH or N63S) all cycled four to five times before becoming irreversibly gelled (Figure 5B, white bars, left panel). In contrast, the ALS/FTD-associated mutant FUS(LC) proteins (S96del or G156E) became irreversibly gelled within two cycles (Figure 5B, gray bars, left panel, p < 0.01, n = 4 replications).

Recombinant FUS assemblies generated in this way displayed biochemical and biophysical characteristics identical to those of FUS assemblies observed in tissues from humans and from our *C. elegans* models. Thus, the ungelled, liquid FUS(LC) domain was fully soluble in RIPA buffer, like wild-type FUS in normal human and *C. elegans* tissues. In contrast, the irreversibly gelled recombinant FUS(LC) domains, regardless of whether generated by multiple cycles of wild-type FUS(LC) or fewer cycles of mutant FUS, were poorly soluble in RIPA buffer but fully soluble in 8 M urea—again like pathological FUS deposits in human and *C. elegans* tissues (Figure 5C, top panel). Furthermore, negative stain electron microscopic (EM) analysis of recombinant irreversible FUS(LC) assemblies revealed a loose meshwork of fibrils, the diameter of which (14.00 ± 1.0 nm, n = 20 fibrils) was indistinguishable from that of fibrils from brain tissue of a human with a FUS-P525L mutation (14.4 ± 3.4 nm, n = 56 fibrils, p = N.S., Figure 5C, bottom panel) (Uchikado et al., 2006). The EM structure for reversible FUS assemblies could not be assessed because their lifetimes in the reversible state (minutes) is shorter than the time required to fix and prepare them for EM analysis. Nevertheless, these observations suggest that the recombinant FUS assemblies modeled here accurately reflect those occurring in vivo in human tissue.

### Single Particle and GFP Tracking Gel Measurements

To further investigate the biophysics of these phase transitions, we used single particle tracking methods (Crocker and Grier, 1996, Rees et al., 2013) to measure changes in the apparent viscosity between liquid/liquid droplet, reversible, and irreversible gel-like FUS states. Briefly, 20-nm radius fluorescent beads were dispersed within a 15-μl volume of FUS solution, which was then cycled through different phases. The Brownian movement of the beads was then used to infer apparent viscosities of the FUS assemblies in each phase. Wild-type FUS(LC) assemblies in liquid state prior to, and immediately after a single round of assembly, displayed very low dynamic viscosity that is typical of a liquid (Figure 5D, left bars: 0.4 ± 0.03 Pa s). When wild-type FUS(LC) formed a reversible gel, there was a modest increase in viscosity (Figure 5D, middle bars: 3.8 ± 0.4 kPa s), which is similar to the viscosity reported for P-granules (∼1.0 Pa s)(Brangwynne et al., 2009). In contrast, mutant FUS(LC) assemblies behaved very differently. In initial liquid state, mutant FUS(LC) had very low dynamic viscosity similar to that of wild-type FUS(LC) ((FUS(LC)(S96del) = 0.04 ± 0.02 Pa s; FUS(LC)(G156E) = 0.013 ± 0.002 Pa s; p = N.S.) (Figure 5D, left bars). However, mutant FUS(LC) that had been assembled once, but was still reversible, displayed a much greater viscosity compared to that of once-gelled wild-type FUS(LC) (FUS(LC)(S96del) = 12 ± 6 kPa s; FUS(LC)(G156E) = 10 ± 1.7 kPa s; p = 0.001) (Figure 5D, middle bars). Even more dramatically, irreversible assemblies, regardless of whether generated using wild-type or mutant FUS(LC), had extremely high viscosities (FUS(LC)(WT) = 15 ± 3 kPa s; FUS(LC)(S96del) = 12 ± 8 kPa s; FUS(LC)(G156E) = 17 ± 2 kPa s, p = N.S.) (Figure 5D, right bars). The very high viscosity values for irreversible FUS assemblies are fully consistent with the electron microscopic structure of these assemblies as loose meshworks of intertwined fibrils that fulfil the IUPAC definition of a hydrogel (Figure 5C). These results support the hypothesis that FUS assemblies can phase-transition between (i) soluble monomeric/liquid droplet like states, (ii) reversible hydrogel-like states, and (iii) irreversible, fibrillar hydrogel-like states.

### Mutant FUS Assemblies Trap Other RNA Binding Proteins

**Figure 6:**
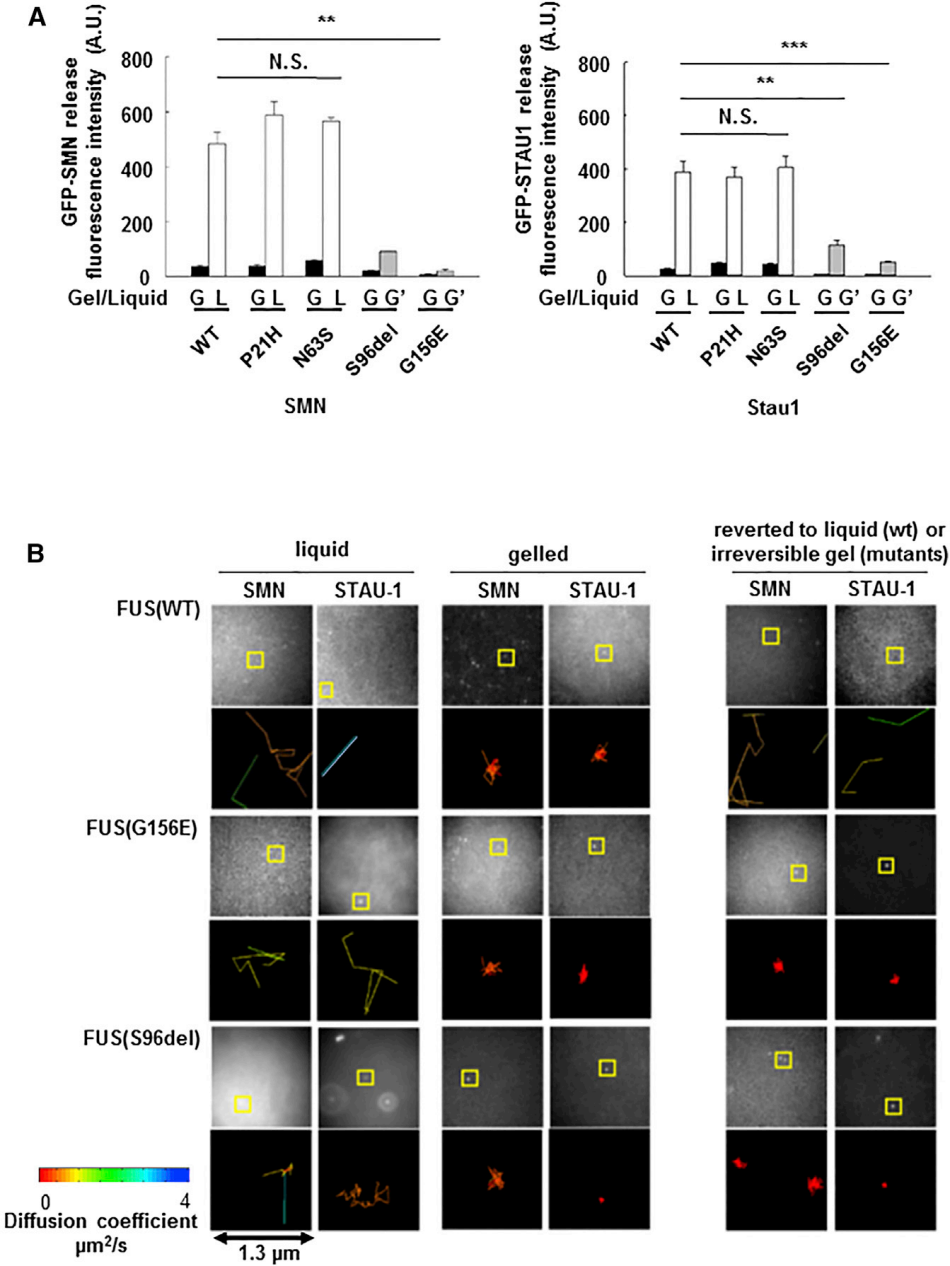
Mutant FUS Forms Poorly Reversible Gels, which Fail to Release Cargo such as SMN and STAU-1 (A) When recombinant GFP-tagged SMN (or STAU-1) protein is mixed with FUS(LC) protein and gel assembly is induced by cooling, little SMN (or STAU-1) is released into the buffer (as measured by fluorescence) regardless of whether the FUS gel is derived from wild-type or ALS/FTD mutant FUS(LC) (black bars). When the gel is reversed by warming to 23°C (white bars), significant SMN (or STAU-1) protein is released into the buffer from FUS(WT) hydrogels. Very little SMN (or STAU-1) is released by the irreversible ALS/FTD mutant FUS(LC) gels (gray bars;^∗∗^p < 0.001). (B) Fluorescent molecule tracking in liquid, reversible gel, and irreversible gel assemblies reveals that in liquid state, SMN and STAU-1 diffuse rapidly at 1–4 μm/s in both mutant and wild-type samples (left two columns). On formation of a gel (center two columns), movement of SMN and STAU-1 is constrained (<1 μm^2^/s). On rewarming, reversible FUS(WT) gels disassemble and release SMN and STAU-1. The irreversible mutant gels continue to constrain movement of SMN and STAU-1. Image stacks are captured at 29 Hz; pixel width corresponds to 160 nm on the specimen. Representative areas are boxed and visualized in a 1.3 μm^2^ plot, color coded to the diffusion coefficient fitted to each track. Error bars are SEM.

We reasoned that condensation of mutant FUS into higher-order assemblies might alter binding and release of RNP granule cargo. To test this idea, we mixed mutant or wild-type FUS(LC) with GFP-tagged SMN (or in separate experiments, with GFP-STAU-1). The mixtures were cooled to induce phase transition and assembly, and buffer was layered on top. The diffusion of SMN (or STAU-1) into the overlying buffer layer was then measured by fluorescence. Very little SMN or STAU-1 was detected in the buffer above any of the gelled assemblies (black bars labeled G-gel in Figure 6A.), regardless of whether the gels were composed of mutant or wild-type FUS(LC). However, SMN and STAU-1 were rapidly released into the buffer following melting of reversible gels compose of wild-type FUS(LC) or non-pathogenic FUS(LC) variants (P21H or N63S) (white bars labeled L-liquid in Figure 6A). However, gels composed of either of the pathogenic mutants (S96del or G156E) failed to revert to liquid states and, as a result, retained SMN and STAU-1 (gray bars labeled G’, Figure 6A). These experiments reveal that cargo proteins can be retained by pathological irreversible FUS assemblies.

To provide further support for the notion that irreversible FUS assemblies might trap cargo RNPs, we used single-molecule imaging to track movement of individual GFP-tagged SMN and GFP-tagged STAU-1 molecules within mutant and wild-type FUS assemblies. Both SMN and STAU-1 moved freely in never-gelled liquid preparations of wild-type and mutant FUS (diffusion coefficients: 1–3 μm^2^/s; Figure 6B, left two columns). Following phase transition and assembly, the diffusion coefficients of both SMN and STAU-1 decreased substantially in reversible assemblies of both wild-type and mutant FUS(LC) (<<1 μm^2^/s) (Figure 6B, center two columns). Fast diffusion of SMN and STAU-1 returned when the reversible FUS(WT) assembly was melted at 23°C (Figure 6B, top right, columns). In contrast, despite re-warming, irreversible FUS assemblies comprised of either twice-cycled mutant FUS or multiply cycled wild-type FUS, continued to significantly constrain movement of SMN and STAU-1 (Figure 6B, bottom right columns).

### Mutant FUS Reduce New Protein Synthesis in Neuronal Axons and Termini

Taken together, our experiments demonstrate that mutant FUS has an increased propensity to form irreversible assemblies that could disrupt the function of FUS-containing RNP granules in several, non-mutually exclusive ways. For instance, irreversible RNP granules containing mutant FUS could disturb the intracellular transport of those RNP granules (Alami et al., 2014, Liu-Yesucevitz et al., 2014). Alternatively, mutant FUS assemblies could trap and titrate out key factors. In the nucleus, this could affect transcription and splicing. In the cytoplasm, and particularly in specific subcellular niches such as dendrites and axon terminals, the trapping could impair local RNA metabolism and translation, thereby converting irreversible FUS RNP granules into local translation repressors. This possibility is distinctly likely because both TIA-1 and mutant FUS are present in stress granules, which act as translational repressors (Anderson and Kedersha, 2002, Bosco et al., 2010, Decker and Parker, 2012).

**Figure 7:**
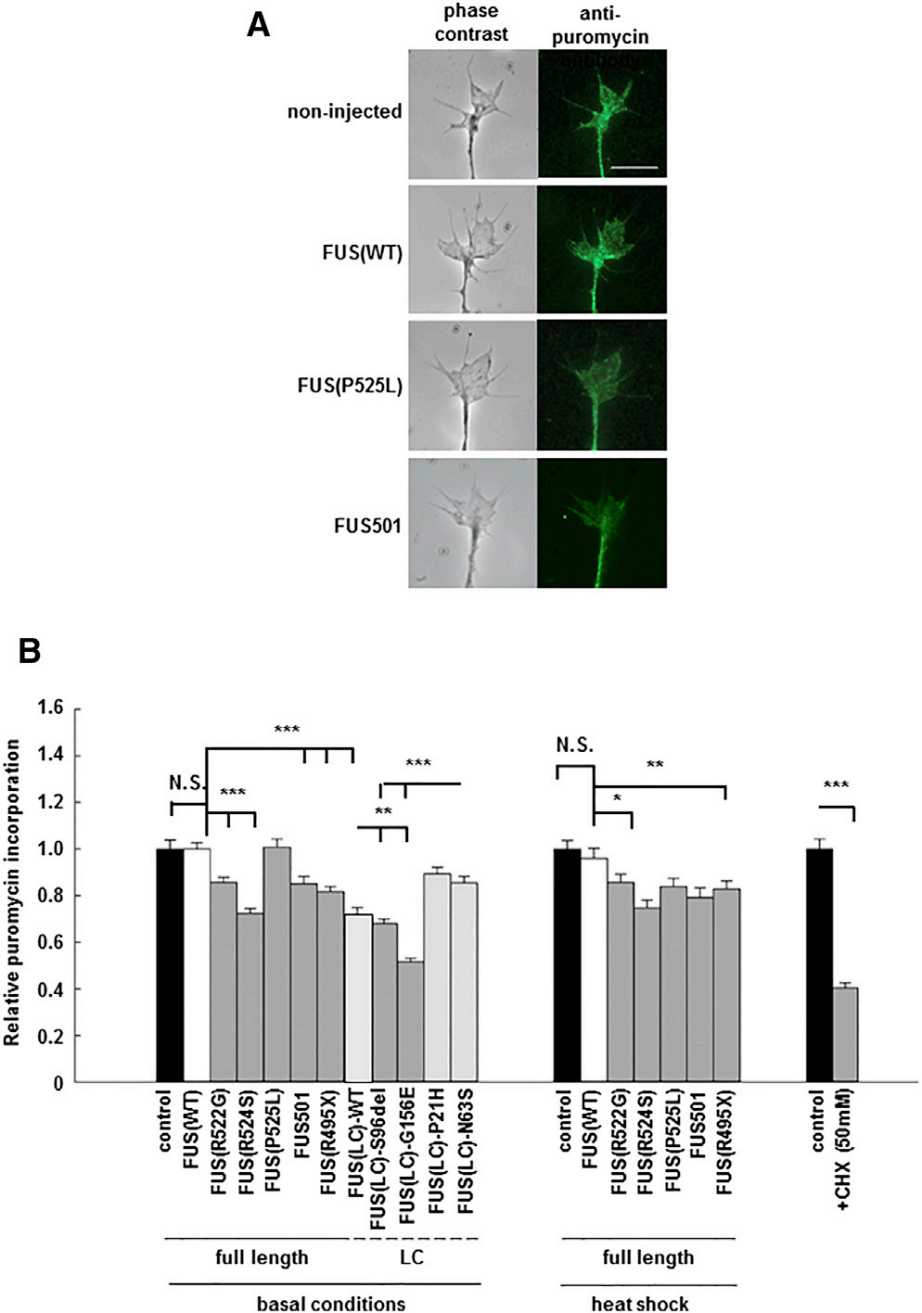
Mutant FUS and FUS(LC) Domain, but Not Full-Length Wild-Type FUS, Cause Reduced New Protein Synthesis When Expressed in Cultured Neurons (A) New protein synthesis was detected by puromycylation in axon terminals of cultured *Xenopus* retinal ganglion neurons (left panel: phase contrast; right panel: anti-puromycin fluorescent images) expressing equivalent quantities of full-length FUS(WT) or various mutant and truncated human FUS proteins. Scale bar, 10 μm. (B) Under basal conditions, de novo protein synthesis is equivalent in non-injected neurons, and in neurons expressing FUS(WT). However, de novo protein synthesis was significantly reduced in neurons expressing ALS/FTD mutants including: FUS(R522G), FUS(R524S), FUS501, FUS(R495X), and in gelling-prone FUS(LC)(WT), FUS(LC)(S96del), FUS(LC)(G156E), FUS(LC)(P21H), and FUS(LC)(N63S). In good agreement with the in vivo studies in *C. elegans*, heat shock causes significant reduction of new protein synthesis in neurons expressing mutant FUS, but minimal impact on non-transfected and FUS(WT) expressing neurons (right panel). N.S., not significant; ^∗^p < 0.05, ^∗∗^p < 0.01, and ^∗∗∗^p < 0.001. Error bars are SEM.

To assess the latter hypothesis, we investigated de novo protein synthesis in axon terminals of primary *Xenopus* retinal neuron cultures expressing equivalent quantities of human full-length FUS(WT), full-length mutant FUS, wild-type FUS(LC), pathogenic mutant FUS(LC), or non-pathogenic FUS(LC) variants (Figure S15). Newly synthesized proteins were detected by pulse labeling with puromycin. The released truncated puromycinylated proteins were imaged and quantified by immunofluorescence using an anti-puromycin antibody (Lin et al., 2009, Schmidt et al., 2009, tom Dieck et al., 2015) (Figure 7A). Under basal conditions, new protein synthesis was significantly reduced in axons expressing full-length FUS(R522G) (0.86 ± 0.02), FUS(R524S) (0.72 ± 0.02), FUS501 (0.85 ± 0.03), and FUS(R495X) (0.82 ± 0.02) (Figure 7B, left panel, p < 0.001). Following heat shock using the identical paradigm employed in *C. elegans*, new protein synthesis was further reduced in all neurons expressing mutant FUS, but not in non-injected neurons or neurons expressing FUS(WT) (Figure 7B, center panel, p < 0.01). This result precisely mirrors the effect of the same heat shock paradigm on in vivo toxicity of mutant FUS in *C. elegans*. Additionally, and again in good agreement with the in vivo neurotoxicity studies in *C. elegans*, new protein synthesis was significantly decreased in neurons expressing FUS(LC), and this was further worsened by the S96del or G156E ALS/FTD mutants (FUS(LC)(WT) (0.72 ± 0.02), FUS(LC)(S96del) (0.68 ± 0.02), and FUS(LC)(G165E) (0.52 ± 0.02), FUS(LC)(P21H) (0.89 ± 0.03), FUS(LC)(N63S) (0.86 ± 0.03), Figure 7B, left panel, p < 0.001). Treatment of neurons expressing wild-type or mutant FUS with cyclohexamide was used as a positive control (Figure 7B, right panel).

Our interpretation of these results is that mutant FUS assemblies perturb RNP granule function by trapping key components of the RNA metabolism and translation machinery. However, an alternate possibility is that the inhibition of new protein synthesis arises from cellular stress due to accumulation of misfolded FUS proteins. While there are several non-specific forms of stress that might differentially occur in animals and cells expressing mutant FUS, the type of stress most clearly associated with the accumulation of misfolded proteins and with inhibition of new protein synthesis is the unfolded protein response (UPR), also known as the endoplasmic reticulum (ER) stress response. Traditionally, the UPR has only been associated with accumulation of misfolded proteins in the ER. However, in some models of neurodegenerative diseases, intracellular misfolded non-ER resident proteins have also been noted to activate the UPR and attenuate protein synthesis (Moreno et al., 2013, Nishitoh et al., 2002).

To address this possibility, we undertook three experiments: the first two, in vivo in our *C. elegans* model; the third, in cultured *Xenopus* neurons.

**Figure 8:**
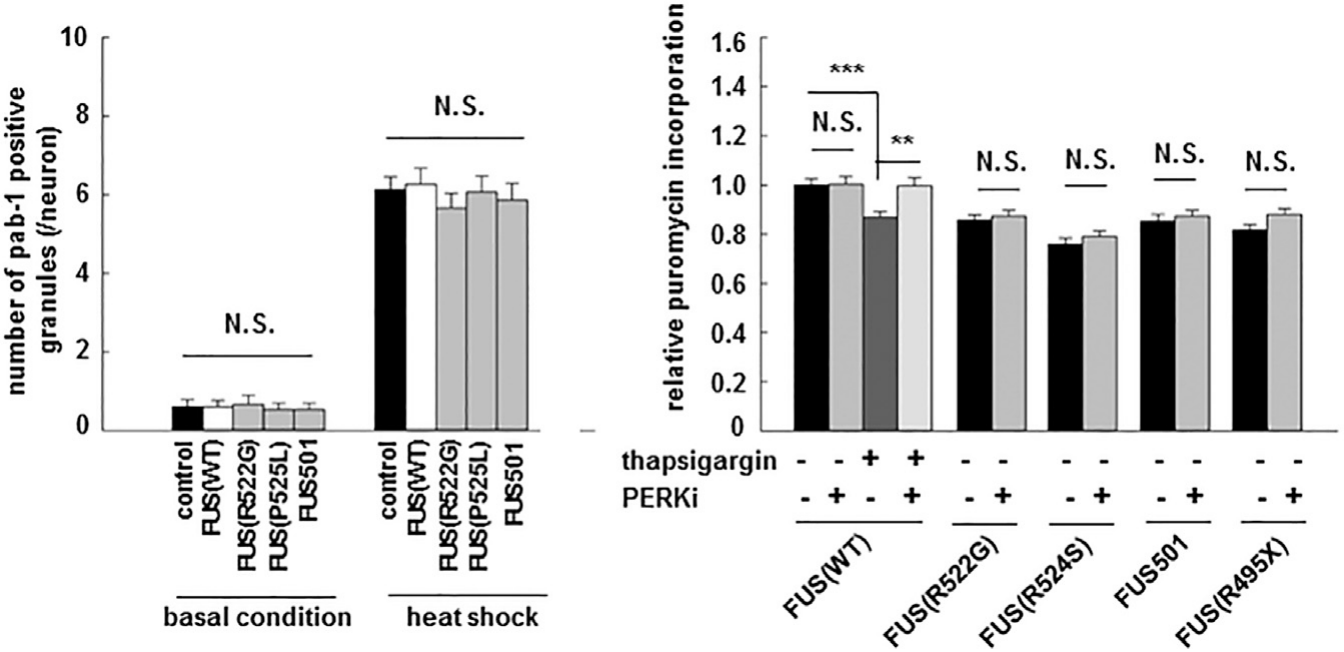
Neurotoxicity of Mutant FUS in *C. elegans* and Reduced New Protein Synthesis in *Xenopus* Neurons Are Unlikely to be Due to Activation of the UPR due to Accumulation of Misfolded FUS (A) In vivo stress granule density measured by counting pab-l-positive granules in *C*. *elegans* under basal conditions and after heat shock were not different between control (empty vector); FUS(WT) and mutant FUS animals, indicating that mutant FUS animals were not under greater unfolded protein stress. (B) Thapsigargin treatment induced UPR and reduced new protein synthesis in *Xenopus* neurons expressing FUS(WT). PERK1 inhibitors rescued reduced protein synthesis in thapsigargin-treated neurons expressing FUS(WT), but not mutant FUS-expressing neurons. Error bars are SEM.

We have previously noted that *C. elegans* expressing either full-length wild-type FUS or full-length mutant FUS had very few intra-cytoplasmic stress granules (zero to two per neuron; p = N.S.) (Murakami et al., 2012). However, animals expressing FUS (WT) and animals expressing mutant FUS are all clearly capable of mounting a vigorous stress response after heat shock, displaying abundant stress granules (five to six granules/cell) (Figure 8, left panel; p = N.S.). These in vivo observations suggest that (1) animals expressing mutant or wild-type FUS are capable of mounting a stress response and (2) mutant-expressing animals are not more stressed by accumulation of misfolded mutant FUS than are animals expressing wild-type FUS.

Second, we examined the activity of the UPR in our *C elegans* model. Translation repression in the UPR is mediated by the eIF2a kinase PERK, and it can be substantially reversed by PERK kinase inhibitors or by PERK deletion. We used RNAi to completely ablate detectable mRNA expression for the endogenous *C. elegans* PERK homolog (pek-1) and the other eIF2a kinase of worms (gcn-2) (Mao and Crowder, 2010, Scheuner et al., 2001). Despite the complete loss of pek-1 and gcn-2, there were no changes in the motor function and lifespan in pek-1 or gcn-2 RNAi-treated mutant FUS-expressing animals (Figures 2A and 2B). These results confirm that neurotoxicity induced by mutant FUS is not directly attributable to activation of PERK (or of eIF2a phosphorylation by other means), and hence, neurotoxicity is unlikely to reflect activation of the UPR.

Third, we undertook PERK inhibitor studies in the *Xenopus* neuron culture model. We compared the effect of the PERK kinase inhibitor GSK2606414 (Axten et al., 2012) on new protein synthesis in neurons expressing (i) wild-type FUS, (ii) wild-type FUS plus thapsigargin (a well-established activator of the UPR), and (iii) ALS/FTD mutant FUS: FUS(R522G), FUS(R524S), FUS501, or FUS(R495X). The UPR was induced, and new protein synthesis was stalled by thapsigargin treatment in neurons expressing full-length FUS(WT) (Figure 8, right panel, lane 3). The UPR was blocked, and new protein synthesis was restored in FUS(WT) neurons treated with GSK2606414 (Figure 8, right panel, lane 4). However, equivalent doses of the inhibitor failed to reverse the impaired new protein synthesis in neurons expressing mutant FUS (Figure 8, right panel, lanes 6, 8, 10, and12).

## Discussion

A detailed explanation of how ALS/FTD-associated mutations in RBPs might impact neuron biology would increase our understanding both of the molecular origins of these neurodegenerative disorders and of the biology of nuclear and cytoplasmic RNP granules. Our work provides several important insights into these issues.

First, the biochemical and biophysical experiments reported here support a physiological model in which reversible phase transition of LC-containing proteins such as FUS can be used to control the assembly and the function of membrane-free structures such as nuclear and cytoplasmic RNP granules. Thus, as we have shown, various cargo elements such as other RNP proteins can be concentrated into a dimensionally constrained space during liquid droplet and reversible hydrogel formation and then released upon their dissolution. The biophysics of conversion from liquid droplet to reversible hydrogel is not yet clear, but they differ only slightly in viscosity, and reversible hydrogels might reflect the partial polymerization into a loose but reversible fibrous network that provides more stability for longer-lived granules.

However, the biophysical propensity of LC-containing proteins to undergo phase transitions to liquid droplets and hydrogels also renders them vulnerable to further condensation into hyper-stable structures. Thus, we have shown that ALS/FTD mutations in FUS dramatically increase the propensity to undergo phase transition into stable “irreversible” fibrillar hydrogel-like states. Indeed, even wild-type FUS can eventually be induced to phase shift into fibrillar hydrogel form—an observation that provides a mechanism for the occurrence of irreversible FUS assemblies in cases of sporadic ALS/FTD with FUS pathology, but without mutations in FUS.

We show that FUS assembly is largely driven by the LC domain. However, our biophysical modeling suggests that other parts of the protein (especially the C terminus) likely buffer this tendency and render the overall protein less prone to form higher order assemblies (Figures S1–S4). Our biophysical modeling also suggests that mutations in the LC domain might directly increase the propensity of the LC domain to form irreversible gels, while mutations in the C terminus are likely to indirectly increase the risk of phase transition by reducing the anti-assembly property of the C terminus (Figures S1–S4). Still other mutations may affect this process by altering binding of RNA or other proteins, which are known to nucleate FUS assembly (Schwartz et al., 2013).

Our work suggests that stable fibrillar hydrogels induce toxicity by mechanisms different from conventional amyloids, namely by disrupting RNP granule function. This conclusion is supported by the fact that downregulating the expression of other RNP granule components destabilizes the pathological RNP granules and rescues toxicity.

Perturbation of RNP granule function could induce neurotoxicity through several mechanisms. Our results strongly suggest that one, but not necessarily the only, neurotoxic effect of phase transition into irreversible higher order assemblies is to disrupt the function of cytoplasmic RNP granules, thereby reducing new protein synthesis in intracellular compartments, such as axon terminals, that are heavily dependent on local translation control by RNP granules (Akbalik and Schuman, 2014, Holt and Schuman, 2013, Jung et al., 2014). This effect would be predicted to be most severe in dendrites and axon terminals of long projection neurons (e.g., motor neurons affected in ALS). The reduction in local new protein synthesis could arise through several molecular mechanisms that are not mutually exclusive, including: altering intracellular transport of FUS granules (Alami et al., 2014, Liu-Yesucevitz et al., 2014), and, as we have shown here, trapping cargo proteins (e.g., TIAR-1) involved in regulating local RNA metabolism and translation.

It is quite probable that similar phase transitions in nuclear FUS might also induce changes in RNA transcription and splicing, which could contribute to toxicity.

Looking forward, it will be important to catalog the differential RNA and protein components of normal and pathological FUS assemblies in ALS and in FTD and to discover the signaling events that govern their assembly and disassembly. Understanding the basis for these processes may provide an explanation for the different clinical phenotypes induced by FUS assemblies. More importantly, as we have shown, manipulation of molecular components of pathological RNP granules (like STAU-1 and SMN) to destabilize pathological assemblies and manipulation of the signaling pathways that govern assembly and disassembly of physiological and pathological RNP granules might be tractable therapeutic targets for these diseases.

The observations reported here are likely to pertain to neurodegenerative diseases associated with *TDP-43*, *TAF15*, *EWS*, *hnRNPA1*, and *B2*. Impaired degradation and removal of these assembly-prone proteins could also explain the occurrence of pathological assemblies of these proteins (particularly TDP-43) in ALS/FTD caused by mutations in autophagy pathway genes such as *p62*, *optineurin*, *TBK-1*, and *valosin-containing protein-1*.

## Experimental Procedures

### Transgenic FUS animals

*C. elegans* stably expressing FUS(WT), FUS(R522G), FUS(R524S), FUS(P525L), FUS(R495X), and FUS501 were generated as described (Murakami et al., 2012). FUS501 mimics human pathogenic truncation mutants such as FUS G497AfsX527(Yan et al., 2010), K510WfsX517X (Yan et al., 2010), G472X (Hara et al., 2012), G466VfsX15 (DeJesus-Hernandez et al., 2010), and R495X(Bosco et al., 2010, Yan et al., 2010) G504Wfs (Kent et al., 2014). Strains lacking the LC motif or expressing LC motif alone (wild-type, S96del and G156E) were made similarly. Strains had similar levels of transgene expression. *C. elegans* hsp-70, pab-1, stau-1, smn-1, tiar-1 and hrp-1 cDNAs were injected into lin-15 animals together with FUS constructs to generate double transgenics.

### Animals and Human Tissues

Experiments on *C. elegans* and *Xenopus* were conducted using approved protocols at the University of Toronto and University of Cambridge. Post-mortem human brain and spinal cord tissues were obtained from four unrelated subjects with FUS(P525L) using institutionally approved protocols at Mayo Clinic (D. Dickson) and Columbia University (N. Shneider).

### Thrashing Assay, Heat Shock Treatment, and Small Molecule Compound Treatment

Motor activity was assessed by counting body bending for 1 min in 4-day-old adult *C. elegans* (Gao and Zhen, 2011). Heat shock involved incubation at 30°C for 6 hr, and then at 22°C, repeated daily for 3 days (Murakami et al., 2012). Scyllo-inositol (100 μM, IO631, TCI America) was added to the media.

### Electron Microscopy

Anterior horns of formalin-fixed spinal cord were removed, infiltrated, and embedded in LR White resin (Polysciences). Sections were stained with 2% uranyl acetate and lead citrate and examined with a Philips 208S electron microscope. Recombinant FUS(LC) was diluted to 0.2 mM, blotted onto Formvar grids, stained with 1% phosphotungstic acid (PTA, pH 7.0), and examined with a Hitachi 7000 electron microscope at 75 kV (Morita et al., 2009).

### Live Cell Imaging

Human Neuroblastoma cell line, SH-SY5Y, was transfected using Neon Transfection System (Life Technologies). After 12 hr incubation, cells were stained and visualized with 710 Zeiss confocal microscope.

### RNAi Assay of *C. elegans* Strains

RNAi was performed as previously (Dixon et al., 2006, Kamath et al., 2003).

### Sequential Fractionation and Western Blot Analysis

Animals were lysed in ten volumes (v/w) of 50 mM Tris-HCl, 750 mM NaCl, 10 mM NaF, and 5 mM EDTA (pH 7.4) with sonication. The lysate was centrifuged and supernatant was collected (high-salt-soluble fraction). The procedure was repeated sequentially in RIPA buffer, 2% SDS buffer, and 8 M urea buffer as described (Murakami et al., 2012).

### RNA Granule Colocalization and GFP Intensity Measurement

Cytoplasmic RFP-positive, GFP-positive, and double-positive granules were counted in tail neurons of double transgenic animals co-expressing GFP-tagged-FUS and RFP-tagged stau-1, smn-1, tiar-1, or hrp-1.

### Single Particle Tracking Analysis in FUS Droplets and Gels

Samples were imaged on a Nikon TE-300 inverted wide-field fluorescence microscope and laser illumination (Cobalt, 488 nm wavelength laser attenuated to 2 mW/cm^2^ intensity on the specimen for imaging GFP, and Toptica iBeam smart 640 nm laser at 20 mW/cm^2^ for imaging fluorescent spheres). Images were captured from a focal plane 15 μm above the coverslip to ensure the observations measured the particles in the bulk specimen and not any adhering to the coverslip. Particle track analysis was performed as described (Crocker and Grier, 1996, Rees et al., 2013). Each repeat measurement of viscosity was obtained from a separate specimen, and at least four image stacks were measured for each.

### Expression, Purification, and Gelling of Recombinant FUS LC Domain Constructs

cDNAs for FUS LC residues 2–214 and its variants were cloned (pOPINS vector) and expressed in *E. coli* BL21(DE3), purified on Ni-NTA resin, buffer exchanged into either droplet buffer (50 mM Tris and 150 mM NaCl [pH 7.5]) or gelation buffer (50 mM Tris and 500 mM NaCl [pH 7.5]). For gelling experiments, FUS samples were concentrated to 1 mM, transferred to thin-walled PCR tubes and incubated at 4°C, and transferred to 23°C to induce melting. For liquid droplet assays, samples were adjusted to 1–5 μM FUS, and 20 μl aliquots were spotted onto siliconised coverslips, cooled to 4°C, and imaged at 40× on an EVOS FL microscope.

### GFP-SMN/STAU-1 Release Fluorescence Assay

Human SMN or STAU-1 with N-terminal His_8_-GFP tag were expressed and purified as for FUS constructs, including a gel filtration step. 5 μl of 10 μM GFP-SMN or GFP-Stau1 protein was mixed with 100 μl of 1 mM wild-type or mutant FUS LC and then incubated at 4°C until gelling occurred. Gels were overlaid with 100 μl of 50 mM Tris with 500 mM NaCl (pH 7.5) buffer; transferred to 23°C to melt; and monitored by fluorescence for release of GFP-SMN or GFP-Stau1.

### Retinal Cultures and Puromycin Labeling

*Xenopus laevis* embryos were fertilized in vitro and raised in 0.1× Modified Barth’s Saline at 14°C–18°C. Capped mRNAs of wild-type or mutant FUS were made (SP6 kit (Ambion)), polyadenylated using Poly(A)-tailing kit, and injected into the two dorsal blastomeres at four-cell stage. Eye primodia from stage 24–26 embryos were dissected and cultured in 60% L-15 on laminin-coated coverslips at 20°C for 48 hr (basal condition), or at 20°C for 42 hr followed by 6 hr at 30°C (heat shock treatment). For puromycin labeling, cultures were treated with 1.8 μM puromycin for 15 min, labeled with Alexa Fluor 488 conjugated anti-puromycin antibody (1:250, Millipore), and imaged as described (Lin et al., 2009, Schmidt et al., 2009, tom Dieck et al., 2015). The fluorescent intensities of 80–250 growth cones per sample were collected. Experiments were performed in three to four replicates.

### Statistical Analysis

Unpaired Student’s t tests, Fisher’s exact test, and ANOVA were performed with SigmaPlot version 11.0.

## Author Contributions

T.M., A.M., S.-P.Y., R.R.F, Y.W., and M.Z. performed the *C. elegans* experiments. S.Q., A.R.C., R.B.D., D.K.-V., Y.L., W.M., and L.D. performed the recombinant protein experiments. E.R., G.S.K.S., F.T.S.C., and C.H.M. did the single particle experiments. G.G.T., G.F., and M.V. did bioinformatics and computational analyses. W.-L.L., D.W.D., and P.E.F. did to the electron microscopy. J.Q.L. and C.H. did the new protein synthesis experiments. P.S.G.-H., M.V., C.H., P.E.F., N.A.S., M.Z., C.F.K., E.R., G.S.K.S., D.R., and D.W.D. designed the experiments. All authors contributed to the writing of the manuscript.

## Supplemental Information

Document S1. Figures S1–S15

Document S2. Article plus Supplemental Information

## References

[bib1] Akbalik G., Schuman E.M. (2014). Molecular biology. mRNA, live and unmasked. Science.

[bib2] Alami N.H., Smith R.B., Carrasco M.A., Williams L.A., Winborn C.S., Han S.S., Kiskinis E., Winborn B., Freibaum B.D., Kanagaraj A. (2014). Axonal transport of TDP-43 mRNA granules is impaired by ALS-causing mutations. Neuron.

[bib3] Anderson P., Kedersha N. (2002). Visibly stressed: the role of eIF2, TIA-1, and stress granules in protein translation. Cell Stress Chaperones.

[bib4] Axten J.M., Medina J.R., Feng Y., Shu A., Romeril S.P., Grant S.W., Li W.H., Heerding D.A., Minthorn E., Mencken T. (2012). Discovery of 7-methyl-5-(1-[3-(trifluoromethyl)phenyl]acetyl-2,3-dihydro-1H-indol-5-yl)-7H-pyrrolo[2,3-d]pyrimidin-4-amine (GSK2606414), a potent and selective first-in-class inhibitor of protein kinase R (PKR)-like endoplasmic reticulum kinase (PERK). J. Med. Chem..

[bib5] Bosco D.A., Lemay N., Ko H.K., Zhou H., Burke C., Kwiatkowski T.J., Sapp P., McKenna-Yasek D., Brown R.H., Hayward L.J. (2010). Mutant FUS proteins that cause amyotrophic lateral sclerosis incorporate into stress granules. Hum. Mol. Genet..

[bib6] Brangwynne C.P., Eckmann C.R., Courson D.S., Rybarska A., Hoege C., Gharakhani J., Jülicher F., Hyman A.A. (2009). Germline P granules are liquid droplets that localize by controlled dissolution/condensation. Science.

[bib7] Cairns N.J., Bigio E.H., Mackenzie I.R., Neumann M., Lee V.M., Hatanpaa K.J., White C.L., Schneider J.A., Grinberg L.T., Halliday G., Consortium for Frontotemporal Lobar Degeneration (2007). Neuropathologic diagnostic and nosologic criteria for frontotemporal lobar degeneration: consensus of the Consortium for Frontotemporal Lobar Degeneration. Acta Neuropathol..

[bib8] Couthouis J., Hart M.P., Shorter J., Dejesus-Hernandez M., Erion R., Oristano R., Liu A.X., Ramos D., Jethava N., Hosangadi D. (2011). Feature Article: A yeast functional screen predicts new candidate ALS disease genes. Proc. Natl. Acad. Sci. USA.

[bib9] Crocker J.C., Grier D.G. (1996). J. Colloid Interface Sci..

[bib10] Decker C.J., Parker R. (2012). P-bodies and stress granules: possible roles in the control of translation and mRNA degradation. Cold Spring Harb. Perspect. Biol..

[bib11] DeJesus-Hernandez M., Kocerha J., Finch N., Crook R., Baker M., Desaro P., Johnston A., Rutherford N., Wojtas A., Kennelly K. (2010). De novo truncating FUS gene mutation as a cause of sporadic amyotrophic lateral sclerosis. Hum. Mutat..

[bib12] Dixon S.J., Alexander M., Fernandes R., Ricker N., Roy P.J. (2006). FGF negatively regulates muscle membrane extension in Caenorhabditis elegans. Development.

[bib13] Dormann D., Capell A., Carlson A.M., Shankaran S.S., Rodde R., Neumann M., Kremmer E., Matsuwaki T., Yamanouchi K., Nishihara M., Haass C. (2009). Proteolytic processing of TAR DNA binding protein-43 by caspases produces C-terminal fragments with disease defining properties independent of progranulin. J. Neurochem..

[bib14] Fang Y.S., Tsai K.J., Chang Y.J., Kao P., Woods R., Kuo P.H., Wu C.C., Liao J.Y., Chou S.C., Lin V. (2014). Full-length TDP-43 forms toxic amyloid oligomers that are present in frontotemporal lobar dementia-TDP patients. Nat. Commun..

[bib15] Gao S., Zhen M. (2011). Action potentials drive body wall muscle contractions in Caenorhabditis elegans. Proc. Natl. Acad. Sci. USA.

[bib16] Gendron T.F., Josephs K.A., Petrucelli L. (2010). Review: transactive response DNA-binding protein 43 (TDP-43): mechanisms of neurodegeneration. Neuropathol. Appl. Neurobiol..

[bib17] Han T.W., Kato M., Xie S., Wu L.C., Mirzaei H., Pei J., Chen M., Xie Y., Allen J., Xiao G., McKnight S.L. (2012). Cell-free formation of RNA granules: bound RNAs identify features and components of cellular assemblies. Cell.

[bib18] Hara M., Minami M., Kamei S., Suzuki N., Kato M., Aoki M. (2012). Lower motor neuron disease caused by a novel FUS/TLS gene frameshift mutation. J. Neurol..

[bib19] Holt C.E., Schuman E.M. (2013). The central dogma decentralized: new perspectives on RNA function and local translation in neurons. Neuron.

[bib20] Jung H., Gkogkas C.G., Sonenberg N., Holt C.E. (2014). Remote control of gene function by local translation. Cell.

[bib21] Kamath R.S., Fraser A.G., Dong Y., Poulin G., Durbin R., Gotta M., Kanapin A., Le Bot N., Moreno S., Sohrmann M. (2003). Systematic functional analysis of the Caenorhabditis elegans genome using RNAi. Nature.

[bib22] Kaminski Schierle G.S., Bertoncini C.W., Chan F.T., van der Goot A.T., Schwedler S., Skepper J., Schlachter S., van Ham T., Esposito A., Kumita J.R. (2011). A FRET sensor for non-invasive imaging of amyloid formation in vivo. ChemPhysChem.

[bib23] Kato M., Han T.W., Xie S., Shi K., Du X., Wu L.C., Mirzaei H., Goldsmith E.J., Longgood J., Pei J. (2012). Cell-free formation of RNA granules: low complexity sequence domains form dynamic fibers within hydrogels. Cell.

[bib24] Kent L., Vizard T.N., Smith B.N., Topp S.D., Vance C., Gkazi A., Miller J., Shaw C.E., Talbot K. (2014). Autosomal dominant inheritance of rapidly progressive amyotrophic lateral sclerosis due to a truncation mutation in the fused in sarcoma (FUS) gene. Amyotroph. Lateral Scler. Frontotemporal Degener..

[bib25] Li Y.R., King O.D., Shorter J., Gitler A.D. (2013). Stress granules as crucibles of ALS pathogenesis. J. Cell Biol..

[bib26] Lin A.C., Tan C.L., Lin C.L., Strochlic L., Huang Y.S., Richter J.D., Holt C.E. (2009). Cytoplasmic polyadenylation and cytoplasmic polyadenylation element-dependent mRNA regulation are involved in Xenopus retinal axon development. Neural Dev..

[bib27] Liu-Yesucevitz L., Lin A.Y., Ebata A., Boon J.Y., Reid W., Xu Y.F., Kobrin K., Murphy G.J., Petrucelli L., Wolozin B. (2014). ALS-linked mutations enlarge TDP-43-enriched neuronal RNA granules in the dendritic arbor. J. Neurosci..

[bib28] Mao X.R., Crowder C.M. (2010). Protein misfolding induces hypoxic preconditioning via a subset of the unfolded protein response machinery. Mol. Cell. Biol..

[bib29] Mitchell S.F., Parker R. (2014). Principles and properties of eukaryotic mRNPs. Mol. Cell.

[bib30] Moreno J.A., Halliday M., Molloy C., Radford H., Verity N., Axten J.M., Ortori C.A., Willis A.E., Fischer P.M., Barrett D.A., Mallucci G.R. (2013). Oral treatment targeting the unfolded protein response prevents neurodegeneration and clinical disease in prion-infected mice. Sci. Transl. Med..

[bib31] Morita M., Osoda K., Yamazaki M., Shirai F., Matsuoka N., Arakawa H., Nishimura S. (2009). Effects of non-steroidal anti-inflammatory drugs on Abeta deposition in Abeta(1-42) transgenic C. elegans. Brain Res..

[bib32] Murakami T., Yang S.P., Xie L., Kawano T., Fu D., Mukai A., Bohm C., Chen F., Robertson J., Suzuki H. (2012). ALS mutations in FUS cause neuronal dysfunction and death in Caenorhabditis elegans by a dominant gain-of-function mechanism. Hum. Mol. Genet..

[bib33] Neumann M., Kwong L.K., Sampathu D.M., Trojanowski J.Q., Lee V.M. (2007). TDP-43 proteinopathy in frontotemporal lobar degeneration and amyotrophic lateral sclerosis: protein misfolding diseases without amyloidosis. Arch. Neurol..

[bib34] Nishitoh H., Matsuzawa A., Tobiume K., Saegusa K., Takeda K., Inoue K., Hori S., Kakizuka A., Ichijo H. (2002). ASK1 is essential for endoplasmic reticulum stress-induced neuronal cell death triggered by expanded polyglutamine repeats. Genes Dev..

[bib35] Rademakers R., Neumann M., Mackenzie I.R. (2012). Advances in understanding the molecular basis of frontotemporal dementia. Nat. Rev. Neurol..

[bib36] Ramaswami M., Taylor J.P., Parker R. (2013). Altered ribostasis: RNA-protein granules in degenerative disorders. Cell.

[bib37] Rees C.E., Ajjawi R., Monrouxe L.V. (2013). The construction of power in family medicine bedside teaching: a video observation study. Med. Educ..

[bib38] Robinson J.L., Geser F., Stieber A., Umoh M., Kwong L.K., Van Deerlin V.M., Lee V.M., Trojanowski J.Q. (2013). TDP-43 skeins show properties of amyloid in a subset of ALS cases. Acta Neuropathol..

[bib39] Scheuner D., Song B., McEwen E., Liu C., Laybutt R., Gillespie P., Saunders T., Bonner-Weir S., Kaufman R.J. (2001). Translational control is required for the unfolded protein response and in vivo glucose homeostasis. Mol. Cell.

[bib40] Schmidt E.K., Clavarino G., Ceppi M., Pierre P. (2009). SUnSET, a nonradioactive method to monitor protein synthesis. Nat. Methods.

[bib41] Schwartz J.C., Wang X., Podell E.R., Cech T.R. (2013). RNA seeds higher-order assembly of FUS protein. Cell Rep..

[bib42] Tartaglia G.G., Vendruscolo M. (2008). The Zyggregator method for predicting protein aggregation propensities. Chem. Soc. Rev..

[bib43] Ticozzi N., Ratti A., Silani V. (2010). Protein aggregation and defective RNA metabolism as mechanisms for motor neuron damage. CNS Neurol. Disord. Drug Targets.

[bib44] tom Dieck S., Kochen L., Hanus C., Heumüller M., Bartnik I., Nassim-Assir B., Merk K., Mosler T., Garg S., Bunse S. (2015). Direct visualization of newly synthesized target proteins in situ. Nat. Methods.

[bib45] Uchikado H., Li A., Lin W.L., Dickson D.W. (2006). Heterogeneous inclusions in neurofilament inclusion disease. Neuropathology.

[bib46] van der Zee J., Van Broeckhoven C. (2014). Dementia in 2013: frontotemporal lobar degeneration-building on breakthroughs. Nat. Rev. Neurol..

[bib47] Wolozin B. (2012). Regulated protein aggregation: stress granules and neurodegeneration. Mol. Neurodegener..

[bib48] Yan J., Deng H.X., Siddique N., Fecto F., Chen W., Yang Y., Liu E., Donkervoort S., Zheng J.G., Shi Y. (2010). Frameshift and novel mutations in FUS in familial amyotrophic lateral sclerosis and ALS/dementia. Neurology.

