# Supplementary material for: ALS/FTD Mutation-Induced Phase Transition of FUS Liquid Droplets and Reversible Hydrogels into Irreversible Hydrogels Impairs RNP Granule Function
Source: Neuron. 2015 Nov 18;88(4):678–90. doi: 10.1016/j.neuron.2015.10.030 (PMC4660210; doi:10.1016/j.neuron.2015.10.030)
Supplement: Document S1. Figures S1–S15 [file mmc1.pdf]

Neuron, Volume *88*

Supplemental Information

ALS/FTD Mutation-Induced Phase Transition of FUS

Liquid Droplets and Reversible Hydrogels into

Irreversible Hydrogels Impairs RNP Granule Function

Tetsuro Murakami, Seema Qamar, Julie Qiaojin Lin, Gabriele S. Kaminski Schierle, Eric Rees, Akinori Miyashita, Ana R. Costa, Roger B. Dodd, Fiona T.S. Chan, Claire H. Michel, Deborah Kronenberg-Versteeg, Yi Li, Seung-Pil Yang, Yosuke Wakutani, William Meadows, Rodylyn Rose Ferry, Liang Dong, Gian Gaetano Tartaglia, Giorgio Favrin, Wen-Lang Lin, Dennis W. Dickson, Mei Zhen, David Ron, Gerold Schmitt-Ulms, Paul E. Fraser, Neil A. Shneider, Christine Holt, Michele Vendruscolo, Clemens F. Kaminski, and Peter St George-Hyslop

Supplemental Data inventory

**Supplemental Item**	**Topic**	**Related to Main Text item**	**Comments**
Supplemental Figure 1-10	Biophysical studies of FUS. Correlation of 8M urea soluble FUS with toxicity	Introduction and main Figure 1	• 1-3 : absence of amyloid characteristics<br>• 4: aggregation propensity<br>• 5: correlation of aggregation propensity with disease severity in humans and C. elegans.<br>• 6-10: basal characteristics of transgenic FUS strains (6-8) and detailed data items (9, 10) correlating abundance of 8M urea soluble FUS species with toxicity (lifespan and motor function). Showing that reducing 8M urea soluble species rescues toxicity, while increasing 8M urea soluble species increases toxicity.
Supplemental Figure 11, 12	Demonstration that the low complexity (LC) domain is necessary and sufficient for toxicity	Main Figure 1	• 11: experimental details from experiments using deletion of LC domain to demonstrate decreases in 8M urea and toxicity<br>• 12: experimental details from experiments using overexpression of wild-type or mutant LC domain to demonstrate increases in 8M urea and toxicity.
Supplemental Figure 13, 14	Demonstration that other RNA binding proteins colocalise with FUS in pathological aggregates, and some help stabilise these aggregates	Main Figure 2, 3	• 13, 14: experimental details from experiments using RNAi treatment against SMN and STAU-1.
Supplemental Figure 15	Demonstration that expression levels of human FUS protein in Xenopus retinal neurons are equivalent	Main Figure 7	• 15: western blotting of Xenopus neurons expressing human FUS

Supplemental Figure 1-3: FUS aggregates are not amyloids in vivo or in vitro

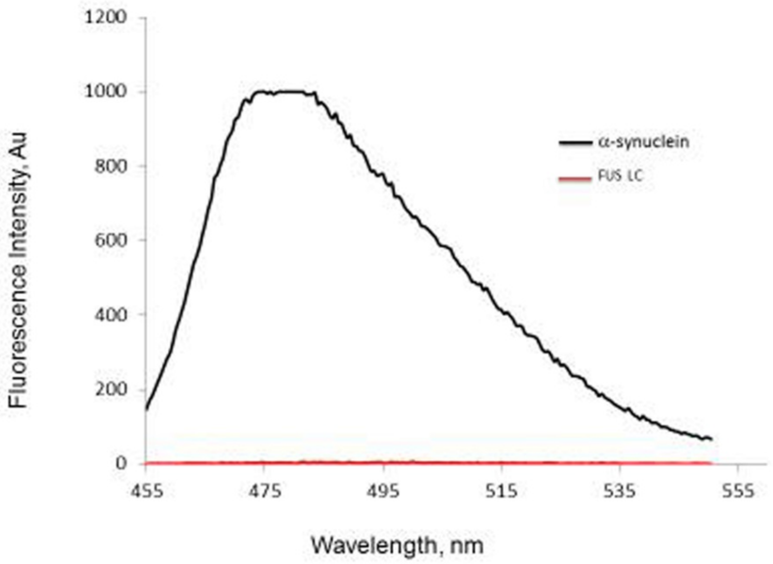

Figure 1: FUS LC hydrogel show no features characteristic of amyloids: No Thioflavin T (ThT) binding: 0.4mg/ml irreversible hydrogel of FUS LC (red) does not bind Thioflavin T. 0.4mg/ml of fibrillar α-synuclein (black) shows fluorescence enhancement upon Tht binding indicating the presence of β-sheet structures characteristic of amyloids. Fluorescence spectra were collected using an LS 55 fluorometer following incubation of gelled proteins samples and α-synuclein with 50 μM ThT. Samples were excited at 442 nm and a fluorescence spectrum collected from 465-520 nm.

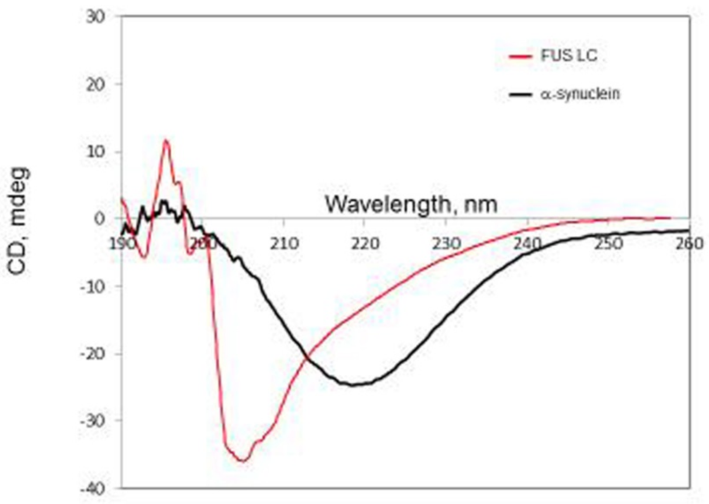

Figure 2: FUS LC hydrogel show no features characteristic of amyloids: Non β-sheet Circular dichroism spectra: FUS LC hydrogel spectra (red) shows no discernable features of the β-sheet secondary structure typical of amyloids. The β-sheet spectra of fibrillar α-synculein (black) is shown for comparison. Circular dichroism spectra were acquired using a Jasco J-810 spectropolarimeter at a scan speed of 50 nm/min with a response time of 2 s and averaged over 20 scans at 25 °C.

<u>In vivo fluorescent life times</u>

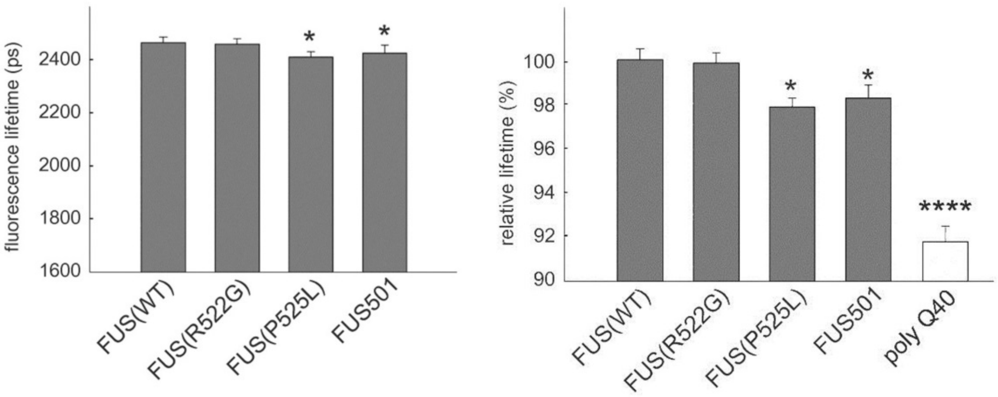

Figure 3: in vivo Mean fluorescence lifetimes are not strongly decreased in FUS gels, but are in conventional amyloid aggregates. Fluorescence lifetimes were measured *in vivo* in wild-type and mutant FUS animals plotted as percentage of GFP-only containing control (blue bars). FUS(P525L) and FUS501 mutant animals are significantly different to

FUS(WT) animals (mean lifetime ± SEM, ANOVA with Scheffe's post hoc test).*$P < 0.05$. In comparison, YFP-labelled classical amyloids such as α-synuclein (not shown) and polyQ40 display >20% fluorescence lifetime drops between soluble and aggregated states (2 way ANOVA, Tukey's multiple comparisons ****$P < 0.0001$).

Supplemental Figure 4: predicted aggregation propensity of FUS holoprotein

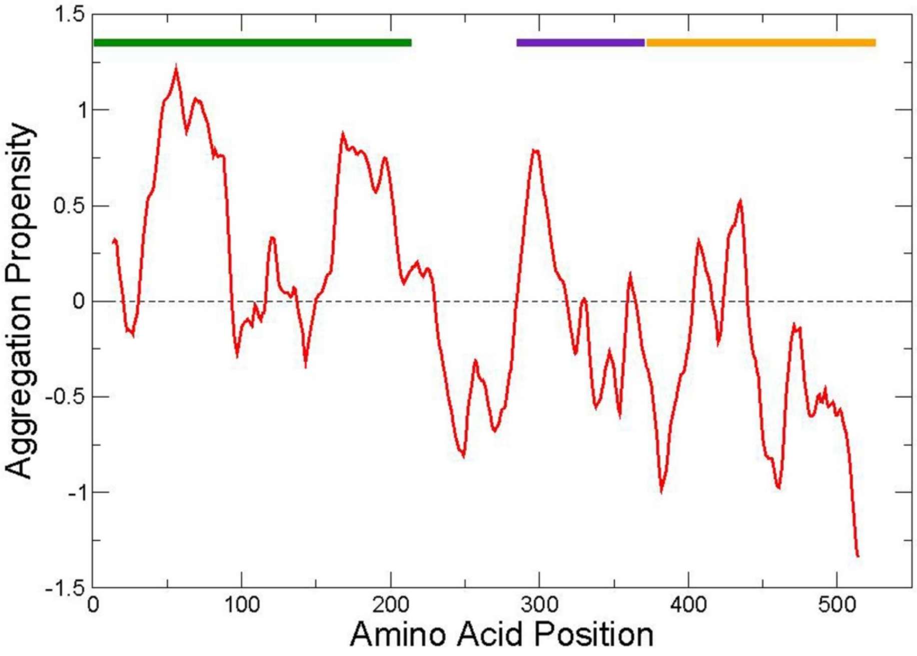

Figure 4. Aggregation propensity profile of FUS (UniProt code: P35637) calculated with the Zyggregator method[1]. The horizontal bars indicate structurally disordered regions located at positions 1-214 (low complexity domain, Gln/Gly/Ser/Tyr-rich; green) and 371-526 (Arg/Gly-rich; orange), as well as the RNA-binding domain at position 285-371 (RRM; purple). The N-terminus shows higher aggregation propensity (residues 1-100) than the C-terminus (residues 450-526). The intrinsic profile is smoothed using a 25-residue window.

Aggregation propensity predictions

The Zyggregator score computes the intrinsic aggregation propensity of proteins, i.e. the propensity of aggregation in the unfolded state[1]. The aggregation propensity score is calculated using the position-dependent score p_i^{agg}. For a given residue *i*, the p_i^{agg} value is calculated as

$$p_i^{agg} = \alpha_h p_h + \alpha_s p_s + \alpha_{hyd} p_{hyd}$$

where p_h and p_s are the propensities for α-helix and β-sheet formation, respectively, and p_{hyd} is the hydrophobicity[1]. The p_i^{agg} values are combined to provide a profile, A_i^p, which

describes the intrinsic propensity for aggregation as a function of the amino acid sequence[2,3]. At each position *i* along the sequence we define the profile A_i^p as an average over a window of seven residues

$$A_i^p = \frac{1}{7}\sum_{j=-3}^{3} p_{i+j}^{agg} + \alpha_{pat} I_i^{pat} + \alpha_{gk} I_i^{gk}$$

where I_i^{pat} is the term that takes into account the presence of specific patterns of alternating hydrophobic and hydrophilic residues[4] and I_i^{gk} is a term that takes into account the gatekeeping effect of individual charges c_i[1]

$$I_i^{gk} = \sum_{j=-10}^{10} c_{i+j}$$

The parameters α are fitted according to the procedure described by DuBay *et al.* [1,2]. In order to compare the intrinsic propensity profiles we normalize A_i^p by considering the average (σ_A) and the standard deviation (σ_A) of A_i^p at each position *i* for random sequences. We thus obtain the normalized intrinsic aggregation propensity profile

$$Z_i^{agg} = \frac{A_i^p - \mu}{\sigma}$$

where we calculated the average μ and the standard deviation σ over random sequences

$$\mu = \frac{1}{(N-8)N_S}\sum_{k=1}^{N_S}\sum_{i=4}^{N-4} A_i^p(S_k) \qquad \sigma^2 = \frac{1}{(N-8)N_S}\sum_{k=1}^{N_S}\sum_{i=4}^{N-4}\left(A_i^p(S_k) - \mu\right)^2$$

In these formulas we used N_S random sequences of length *N*, and we verified that μ and σ are essentially constant for values of *N* ranging from 50 to 1000.

1. Tartaglia, G. G. *et al.* Prediction of aggregation-prone regions in structured proteins. *J Mol Biol* **380,** 425–36 (2008).
2. DuBay, K. F. *et al.* Prediction of the absolute aggregation rates of amyloidogenic polypeptide chains. *J Mol Biol* **341,** 1317–26 (2004).
3. Tartaglia, G. G., Cavalli, A., Pellarin, R. & Caflisch, A. Prediction of aggregation rate and aggregation-prone segments in polypeptide sequences. *Protein Sci* **14,** 2723–34 (2005).
4. Xiong, H., Buckwalter, B., Shieh, H. & Hecht, M. Periodicity of Polar and Nonpolar Amino Acids is the Major Determinant of Secondary Structure in Self-Assembling Oligomeric Peptides. *Proceedings of the National Academy of Sciences* **92,** 6349–6353 (1995).

Supplemental Figure 5: Correlations of aggregation propensity and disease severity

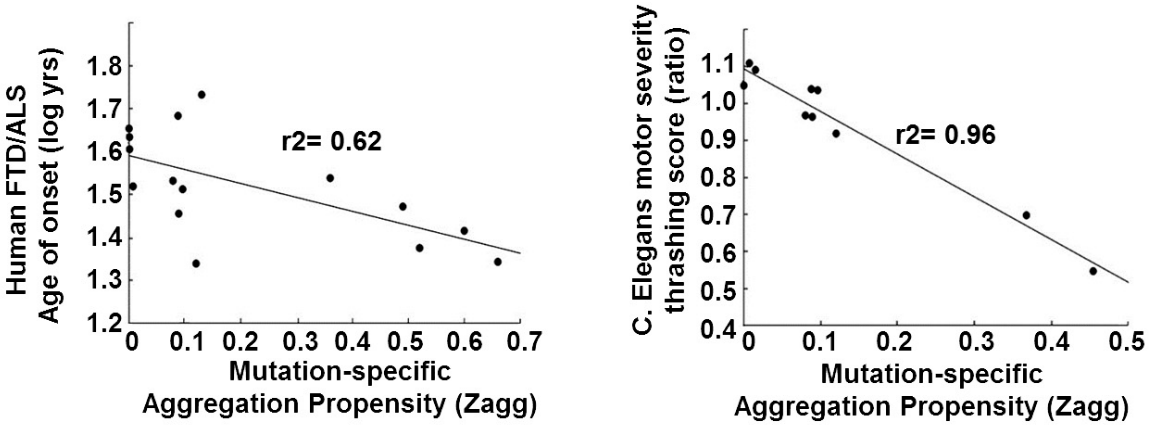

Left panel: The severity of FTLD/MND induced by each FUS mutation (as measured by the age-of-onset) shows a weak correlation with the aggregation propensity of that mutation (rR= 0.62). The weak correlation likely reflects both the effects of the outbred genetic background of human populations, and differences in presentation (bulbar versus limb weakness etc), which profoundly affect the clinical outcome in humans with ALS.

Right panel: The severity of neuronal dysfunction induced by each FUS mutation (as measured by the thrashing score), is strongly correlated with the aggregation propensity of that mutation (r = 0.98).

Supplemental Figure 6: mutant FUS protein mislocalises in the cytoplasm in *C. elegans*.

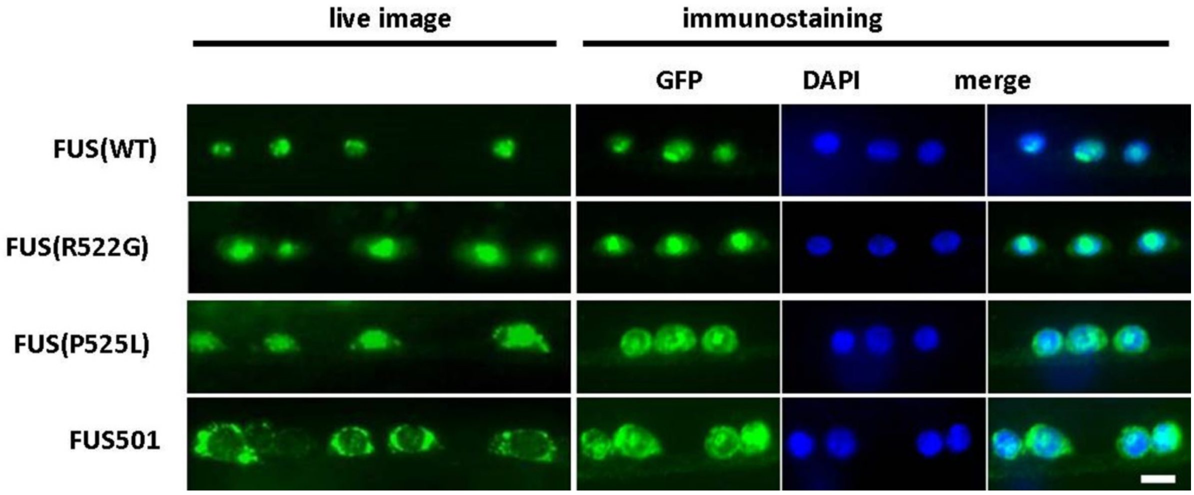

Supplemental Figure 7 and 8: Basal data of wild-type and mutant FUS strains.

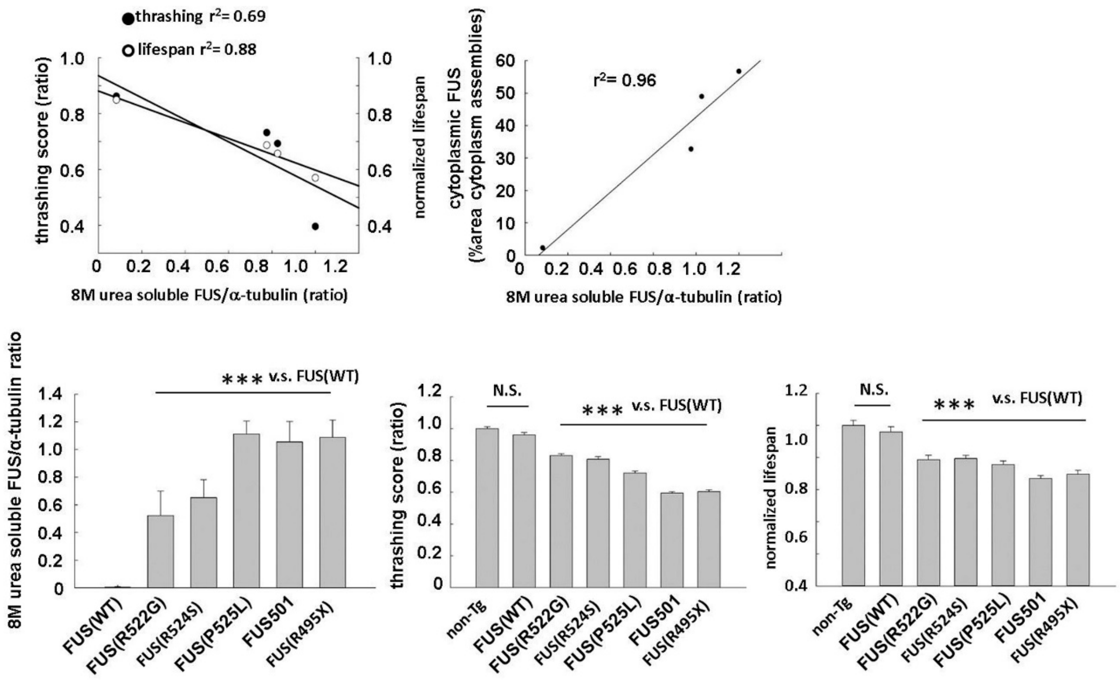

Supplemental Figure 7: **Basal data of Wild-type and mutant FUS strains.**

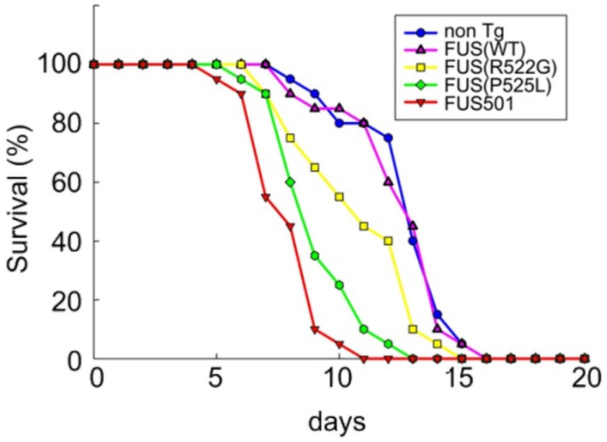

Supplemental Figure 8: Baseline survival curve. All mutant FUS strains show shorter life spans compared to FUS(WT) strain, whereas lifespans of FUS(WT) are not different from N2 strain.

Supplemental Figure 9 and 10: The severity of neurotoxic phenotypes can be increased or decreased by manipulating the abundance of 8M Urea soluble assemblies in *C. elegans*

Repeated mild heat stress induces increased abundance of FUS assemblies in neuronal cytoplasm (not shown) and of 8M urea soluble FUS assemblies in animals expressing mutant FUS but not wild-type FUS. The presence of more FUS assemblies is accompanied by a marked increase in neurotoxicity as manifest by significantly decreased thrashing scores compared to genotypic controls and to wild-type controls and reduced longevity. Note that heat shock induces a mild, non-specific decrease in motor performance in both non-transgenic and FUS(WT) animals. However, this reduction is proportionately much less than the decrements observed in animals expressing mutant FUS. Similarly, although the increase in 8M urea soluble FUS assemblies in the FUS(R522G) animals is sufficient to cause an increase in motor dysfunction, the phenotype in these animals is milder and it does not increase mortality. N.S. = not significant, $^{**}P < 0.01$, $^{***}P < 0.001$. Non-transgenic animals, and animals expressing wild-type or mutant FUS were treated by the addition of a small molecule anti-aggregant (scylloinositol) to the culture plates (grey bars) or buffer (black bars). Scylloinositol had no effect on FUS localisation, 8M urea soluble FUS assembly or motor function and longevity in non-transgenic animals or expressing wild-type FUS. In animals expressing mutant FUS, scylloinositol reduces cytoplasmic FUS (not shown), and reduces the abundance of 8M urea soluble FUS assemblies. This reduction is accompanied by significantly improved motor function, which in the case of FUS (R522G) and FUS(P525L) expressing animals almost approximates the motor performance of FUS(WT) and N2 animals. The reduction in 8M urea soluble FUS assemblies is accompanied by improved lifespan in the FUS(P525L) and FUS501 mutant expressing animals. Scylloinositol had no effect on the already essentially normal lifespan of the mildly affected FUS (R522G) animals. FUS501 animals were crossbred with animals expressing hsp-70. Overexpression of hsp-70 in the FUS501 animals resulted in significant reduction in 8M urea soluble FUS assemblies (3C, left panel) accompanied by significant improvement in motor function (3C, right panel) and longevity (3D). Overexpression of hsp-70 alone had no effect on these parameters. Because of the site of integration of the hsp-70 transgene on the same chromosome as the FUS(WT) and FUS(P525L) transgenes, we were unable to generate double transgenic animals for these lines. N.S. = not significant, $^{**}P < 0.01$, $^{***}P < 0.001$.

Figure 9: Heat Shock increases 8M urea soluble assemblies and neurotoxicity

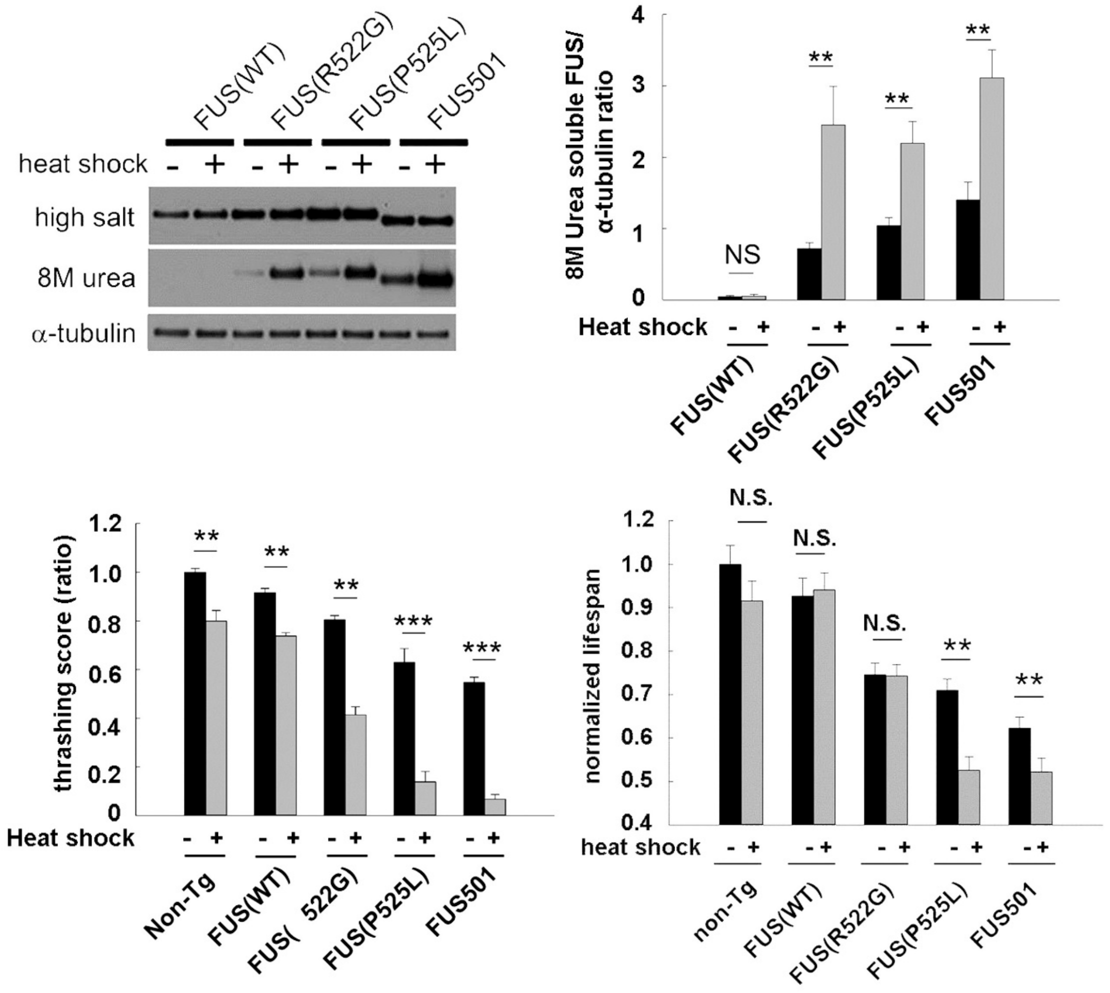

Kaplan-Meier curves for heat-shock

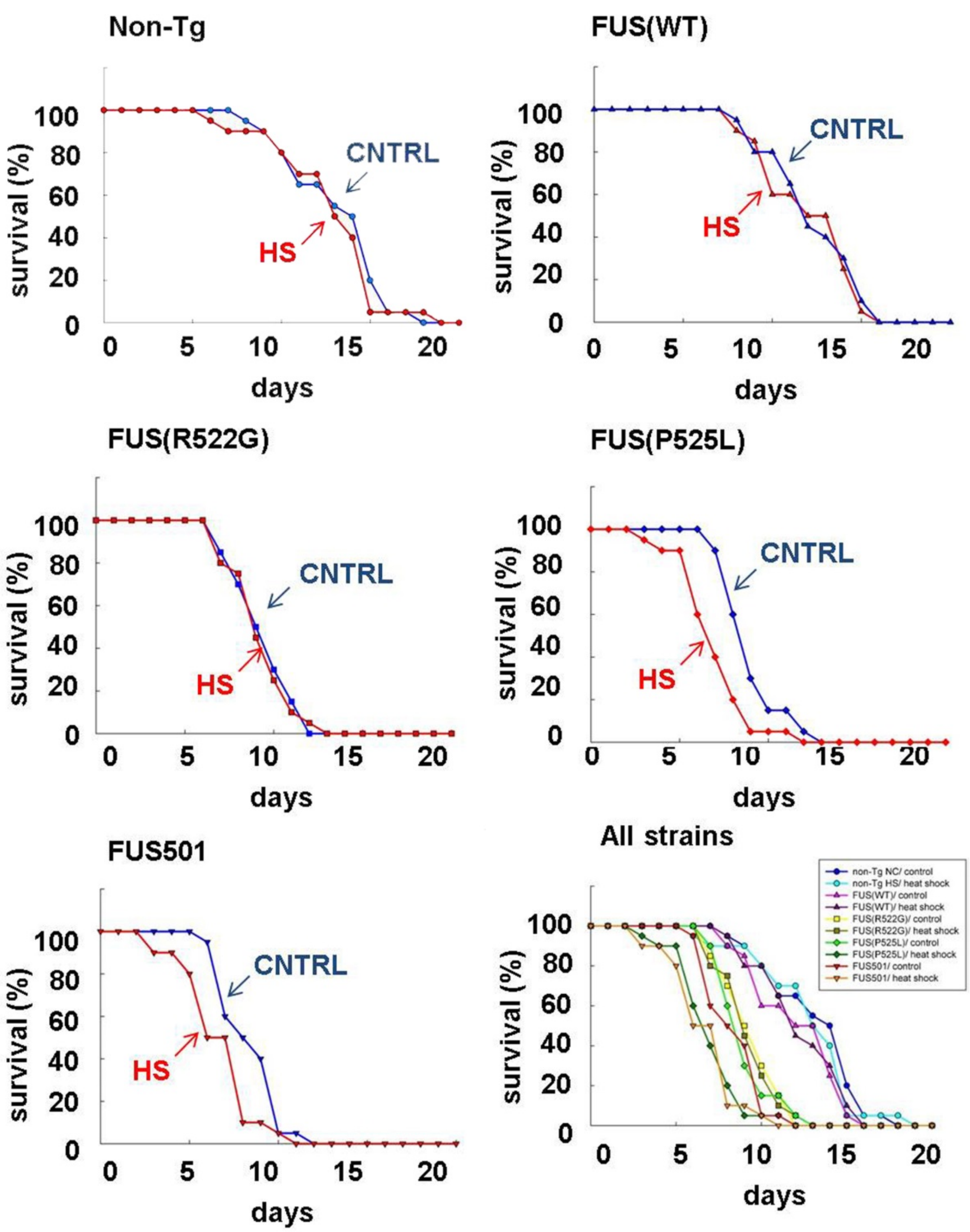

*CNTRL; control, HS; heat shock

Figure 10: Scyllo-inositol and over-expression of HSP70 decrease 8M urea assemblies and reduce neurotoxicity

<u><Scyllo-inositol></u>

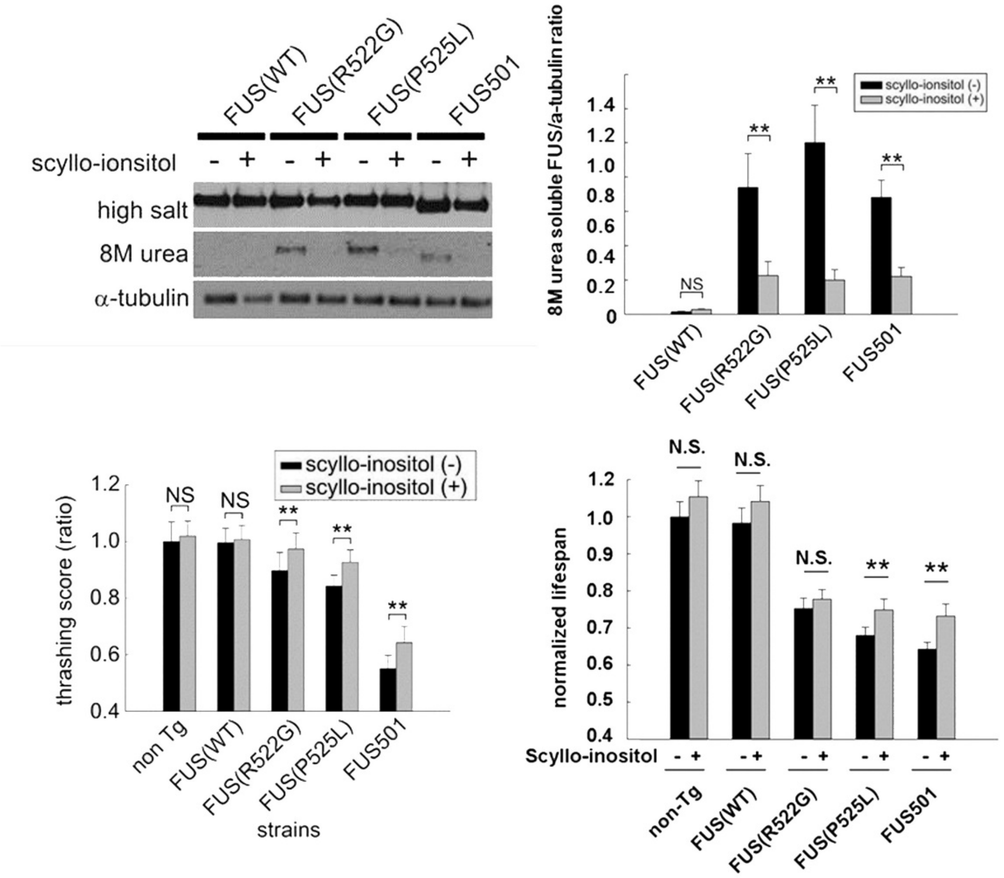

Kaplan-Meier curves for scyllo-inositol

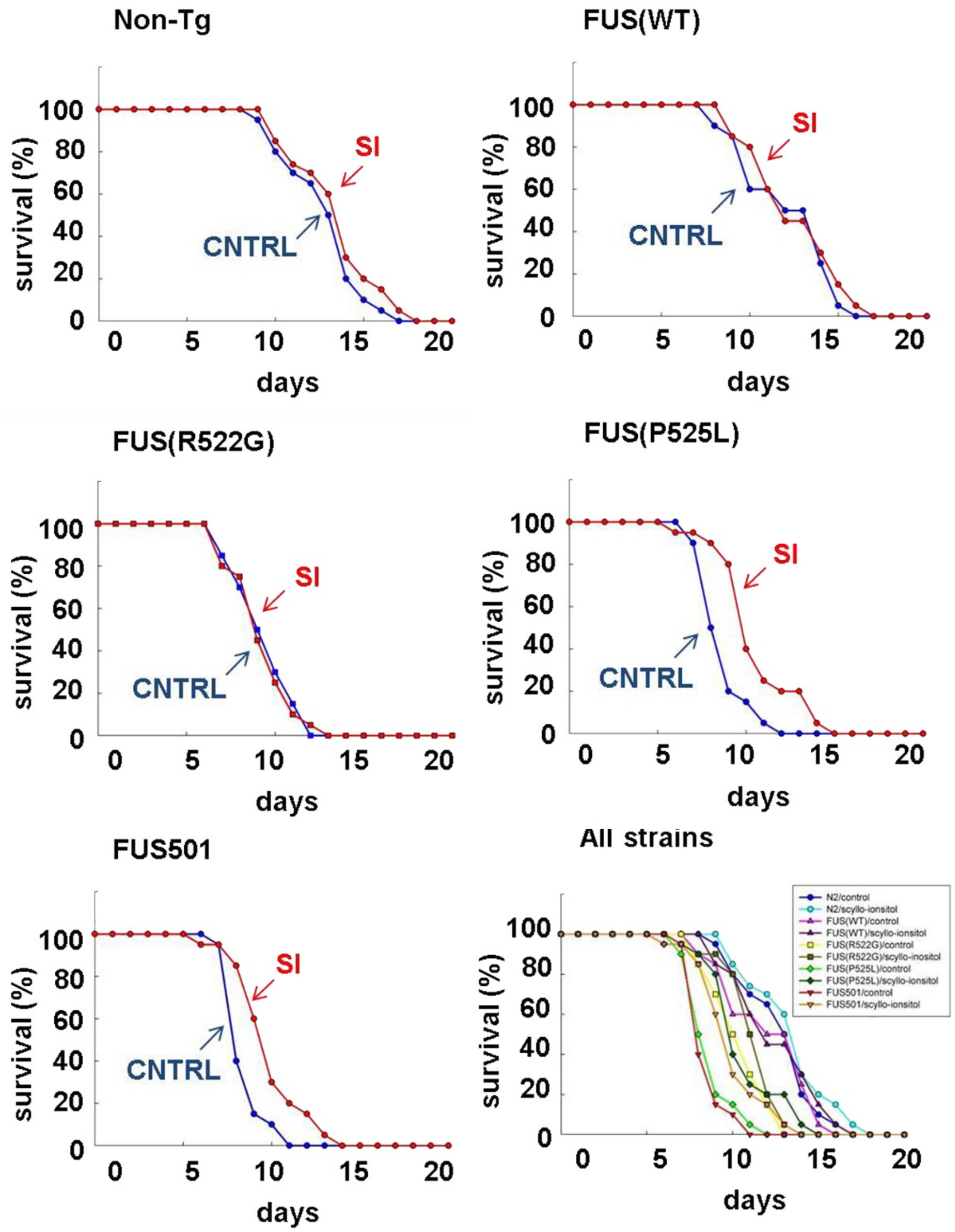

*CNTRL; control, SI; scyllo-inositol

<hsp-70>

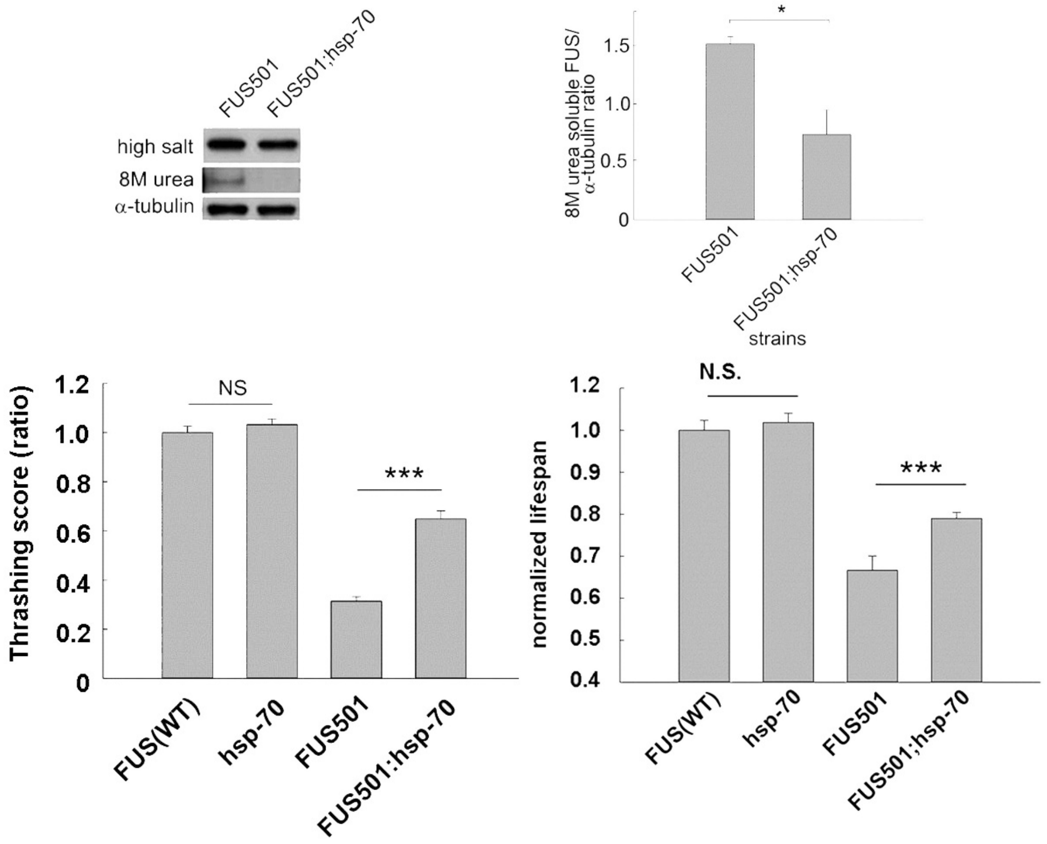

Kaplan-Meier curves for hsp-70

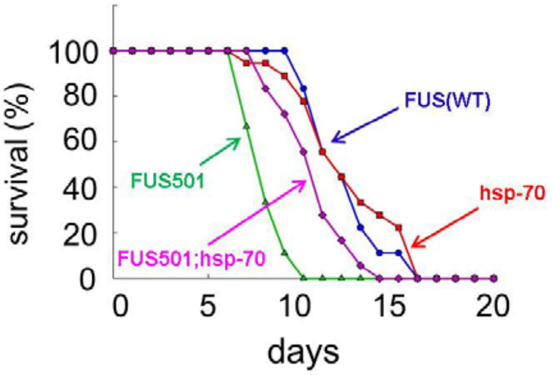

Supplemental Figure 11: Deletion of LC domain decrease 8M urea assemblies and reduce neurotoxicity

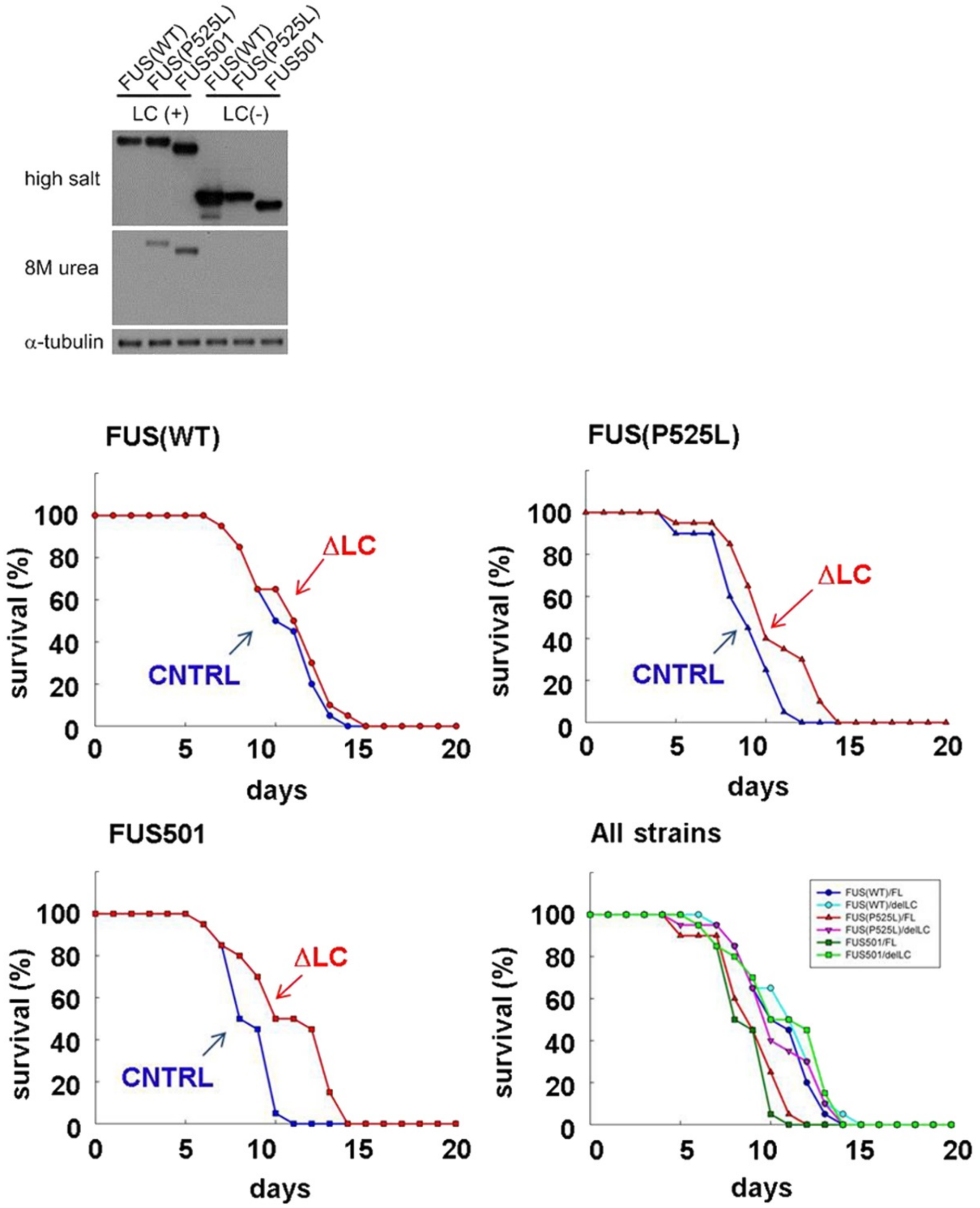

Kaplan-Meier curves for delLC strains

*CNTRL; control, ΔLC; strains overexpressing delLC motif.

Supplemental Figure 12: Overexpresison of LC domain increases 8M urea soluble assemblies and neurotoxicity

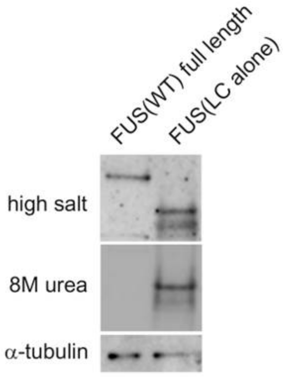

Kaplan-Meier curves for LC mutants

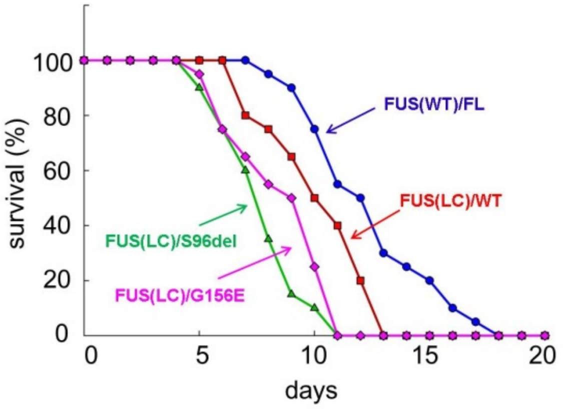

Supplemental Figure 13: smn1 and stau-1 are structural and stabilizing components of mutant pathological FUS granules

Kaplan-Meier curves for smn1 and sta1 RNAi

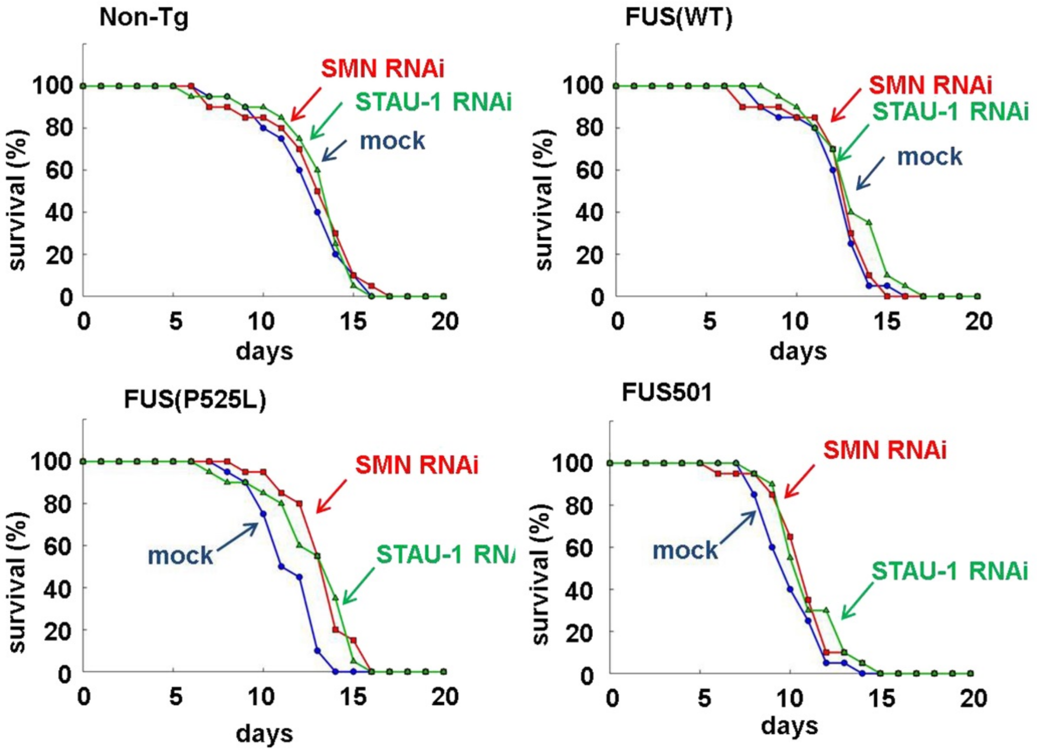

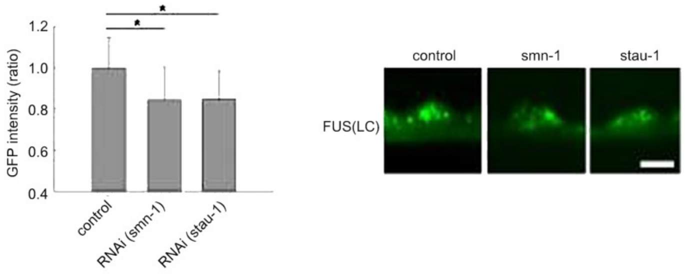

Supplmental Figures 14: SMN, STAU-1 co-localise with pathological mutant FUS aggregates in the cytoplasm of C elegans neurons.

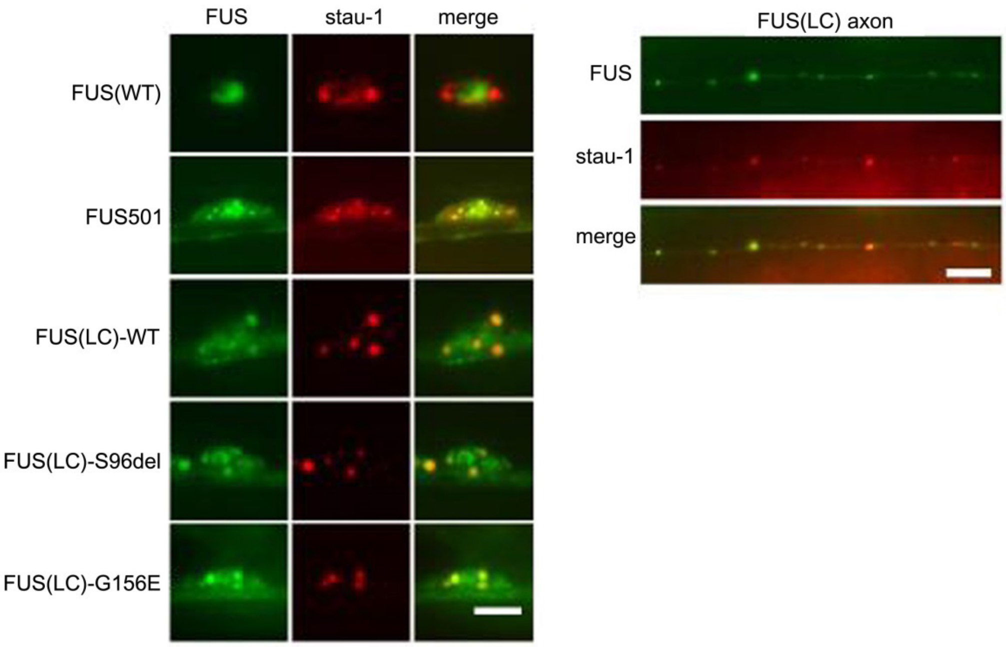

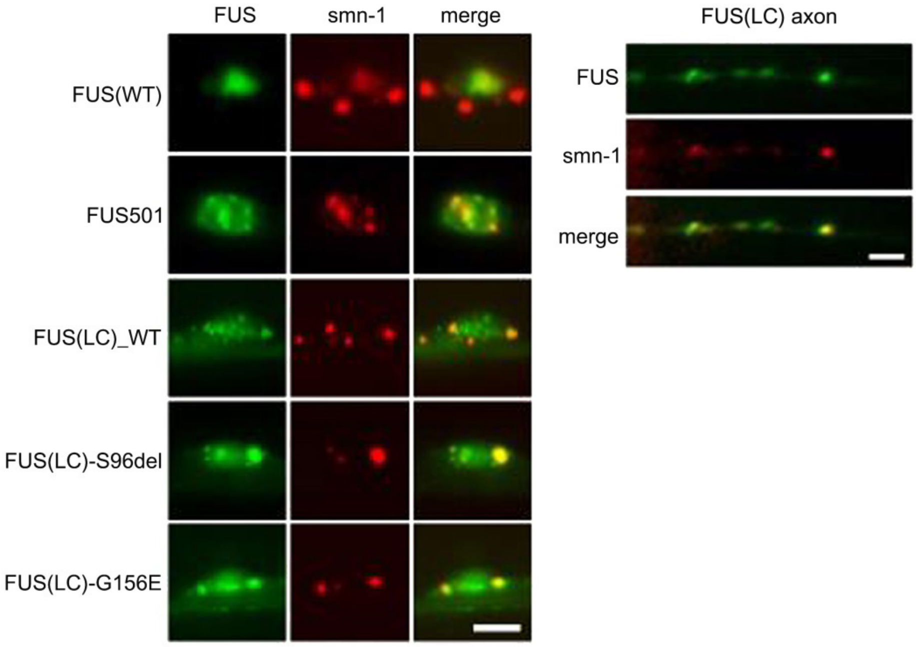

Supplemental Figure 15: Mutant FUs reduces new protein synthesis in neuronal axons and termini.

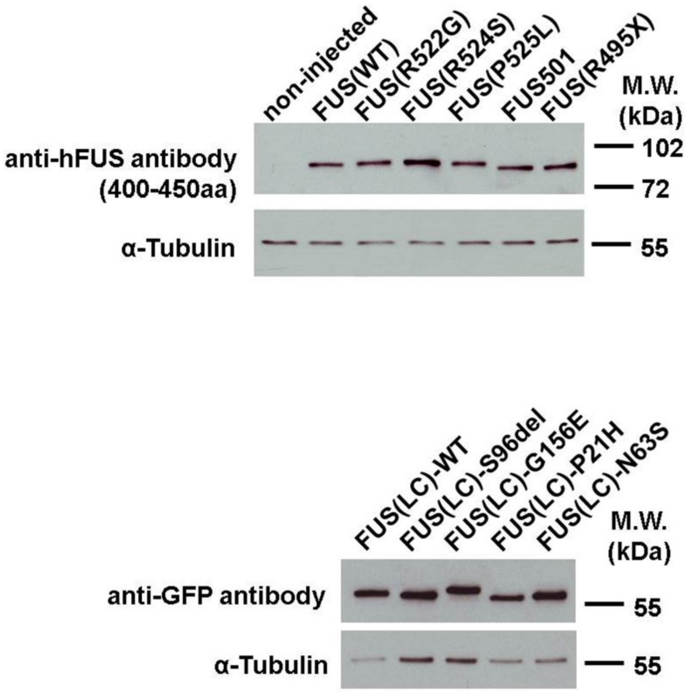

Western blotting of *Xenopus* retinal neurons overexpressing wild-type and mutant FUS proteins.
